# CIDANE: comprehensive isoform discovery and abundance estimation

**DOI:** 10.1186/s13059-015-0865-0

**Published:** 2016-01-30

**Authors:** Stefan Canzar, Sandro Andreotti, David Weese, Knut Reinert, Gunnar W. Klau

**Affiliations:** Center for Computational Biology, McKusick-Nathans Institute of Genetic Medicine, Johns Hopkins University School of Medicine, Baltimore, MD 21205 USA; Department of Mathematics and Computer Science, Institute of Computer Science, Freie Universität Berlin, Arnimallee 14, Berlin, 14195 Germany; Life Sciences, Centrum Wiskunde & Informatica (CWI), Science Park 123, Amsterdam, 1098 XG The Netherlands; Toyota Technological Institute at Chicago, 6045 S. Kennwood Avenue, Chicago, IL 60637 USA

## Abstract

We present CIDANE, a novel framework for genome-based transcript reconstruction and quantification from RNA-seq reads. CIDANE assembles transcripts efficiently with significantly higher sensitivity and precision than existing tools. Its algorithmic core not only reconstructs transcripts ab initio, but also allows the use of the growing annotation of known splice sites, transcription start and end sites, or full-length transcripts, which are available for most model organisms. CIDANE supports the integrated analysis of RNA-seq and additional gene-boundary data and recovers splice junctions that are invisible to other methods. CIDANE is available at http://ccb.jhu.edu/software/cidane/.

**Electronic supplementary material:**

The online version of this article (doi:10.1186/s13059-015-0865-0) contains supplementary material, which is available to authorized users.

## Background

High-throughput sequencing of cellular RNA (RNA-seq) aims at identifying and quantifying the set of all RNA molecules, the transcriptome, produced by a cell. Despite having largely identical genomes, the RNA content of cells differs among tissues, developmental stages, and between disease and normal condition. For eukaryotes, differences are determined by the set of genes being expressed, but also by the different mRNA isoforms each gene may produce; alternative splicing, alternative transcription and polyadenylation define and combine exons in distinct ways.

RNA-seq technology can generate hundreds of millions of short (50–250 bp) strings of bases, called reads, from expressed transcripts at a fraction of the time and cost required by conventional Sanger sequencing. The wealth of RNA-seq data produced recently has revealed novel isoforms [1–3] and new classes of RNA [4], allowed a better characterization of cancer transcriptomes [5, 6], and led to the discovery of splicing aberrations in disease [7, 8].

However, the step from sequencing to profiling the cellular transcriptome involves solving a high-dimensional complex puzzle, which poses major challenges to bioinformatics tools as every single short read carries little information by itself. In particular, repeat and paralogous sequences, as well as low-expressed regions and minor isoforms, are difficult to assemble. Notice that transcripts that are moderately expressed only in a subpopulation of cells manifest an overall low expression level, as might be the case for long noncoding RNAs (lncRNAs) [4].

Unlike de novo transcript assembly approaches, which assemble reads solely based on the overlap of their sequences, genome-based methods employ a high-quality reference genome to resolve better ambiguities imposed by highly similar regions of the genome and to recover lower expressed transcripts. Genome-based methods first align reads to the genome to determine where each of the reads originated and then assemble the alignments into transcript models. This in turn introduces a critical dependence on the accuracy of the read alignment, which is affected by sequencing errors, polymorphisms, splicing, and ambiguous reads that belong to repeats. Reads spanning splice junctions between exons are particularly informative since they provide an explicit signal for the detection of splice donor and acceptor sites. At the same time, the spliced alignment of such reads is computationally challenging and error prone.

For an unbalanced split, the prefix or suffix of a read that spans one of the two consecutive exons may be short and thus aligns equally well to a large number of genomic positions. Guessing the true origin can be further hampered by polymorphisms near the splice site. Besides *incorrect* spliced alignments, this can also lead to *missed* splice junctions, i.e., exon–exon junctions that are not supported (covered) by any spliced alignment. Missed junctions can also result from read coverage fluctuations (biases) or a generally low transcript abundance. While some of the existing methods do take into account incorrect alignments by applying ad hoc filters (Scripture [9] and CLIIQ [10]) or by not requiring the isoform selection model to explain all input alignments (MITIE [11]), none of the existing approaches is able to deal with missed junctions. In this work we present a novel framework CIDANE (comprehensive isoform discovery and abundance estimation), which, for the first time, allows us to recover isoforms with uncovered splice junctions that are invisible to all existing approaches.

On a high level, existing methods for genome-based transcript assembly adhere to the following scheme: First, a set of candidate isoforms is defined as paths in a graph representing the base or exon connectivity as indicated by the aligned reads. Then, a *small* subset of isoforms is selected that explains the read alignments *well*. Since only a small number of transcripts is typically expressed in a given cell type (compared to the number of candidates), the restriction to few isoforms prevents fitting noise in the data.

Current methods mostly differ in the trade-offs they apply between the complexity of the model and the tractability of the resulting optimization problem, which largely determines the quality of the prediction:

1. Since the number of potential isoforms grows exponentially with the number of exons of the locus, all existing methods restrict either implicitly or explicitly the number of candidates they consider. Methods that do not enumerate isoforms explicitly either employ a simplified model with transcript-independent coefficients (e.g., MITIE and Traph [12]) or separate the intrinsically interdependent minimality and accuracy objectives (Cufflinks [2]).
2. A second crucial algorithmic design decision is how to *balance* the two concurrent objectives. In an extreme case, the two objectives are treated independently (e.g., Cufflinks, CLASS [13], CLIIQ, Traph, and IsoInfer [14]). More recent state-of-the-art methods (e.g., MITIE, iReckon [15], SLIDE [16], IsoLasso [17], and StringTie [18]) have recognized the importance of optimizing both objectives simultaneously and balance minimality and accuracy heuristically.
3. Among methods that simultaneously optimize for both objectives, the measure of minimality has an enormous impact on the tractability of the resulting problem. The most immediate measure, the number of predicted transcripts (*L*^0^ norm), leads to non-convex objectives and a computationally intractable optimization problem. Methods like MITIE, StringTie, Montebello [19], and iReckon, which employs a novel non-convex minimality measure, therefore resort to a forward stepwise regression strategy, a Monte Carlo simulation, or numerical optimization combined with random restarts, that generally do not find the best solution in this model. Methods like SLIDE and IsoLasso thus replace the *L*^0^ norm by the convex *L*^1^ norm, i.e., the sum of transcript abundances.
4. Concerning the measure of accuracy, methods apply a least-squares loss function (e.g., IsoLasso, SLIDE, or TRAPH), least absolute deviation (not explicitly modeled in StringTie), or compute more generally a maximum likelihood assignment of reads to candidate isoforms. The latter typically requires a preselection of transcripts (Cufflinks) or leads to the intractability of the resulting problem (iReckon and Montebello).

Here we present CIDANE, a comprehensive tool for genome-based assembly and quantification of transcripts from RNA-seq experiments. The central idea of CIDANE is to trade the ability to determine the provably best transcript prediction in the underlying model for a slight approximation of the loss function. Intuitively, the accuracy and minimality measure (see (3) and (4)) fit noisy observations (read alignments) and thus, the impact of their (adjustable) approximation on the overall prediction performance is expected to be rather limited. CIDANE therefore minimizes a least-squares loss function based on full-length transcripts and replaces the *L*^0^ minimality measure by the convex *L*^1^ norm, which, in fact, *selects* a subset of transcripts with non-zero expression levels that is predicted to be expressed in a given cell type. A formulation based on full-length isoforms enables us to develop a comprehensive linear model (like SLIDE), which, among other things, takes into account the dependence of the distribution of read pairs along a given transcript on the estimated fragment length distribution. In contrast to previous methods, we employ a state-of-the-art machine-learning algorithm to compute the optimal balance (according to a strict mathematical measure) between accuracy and minimality at essentially no additional computational cost. In a second phase, CIDANE linearizes the least-squares loss function with bounded error, which allows us to formulate our model based on all possible candidate transcripts, including transcripts with uncovered splice junctions, without having to enumerate them explicitly. Following the principle of *delayed column generation* [20], we only add isoforms to our model on demand, i.e., if they help to strictly improve the overall prediction.

**Fig. 1:**
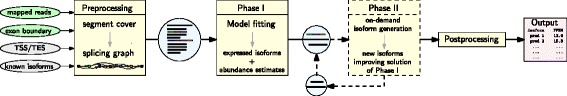
General work flow of CIDANE. Mandatory inputs (mapped RNA-seq reads and exon boundaries) and optional inputs (TSS, TES, and known transcripts) are used to summarize read alignments into segment covers, which count reads falling into non-ambiguously spliced segments of genes. From the corresponding splicing graph representation [37], an initial set of candidate isoforms is derived and a subset of expressed isoforms with estimated abundances is predicted by a regularized regression method during phase I. This set forms the input to the optional phase II, where improving isoforms are built on demand by a delayed column generation approach. New candidates inferred in phase II are then added to the initial candidate set to achieve a better fit of the model. After re-estimation of abundances and filtering (post-processing), a list of isoforms with abundance estimates is returned in gtf format

CIDANE implements a design that separates the assembly of full-length transcripts from the identification of elementary components, i.e., exons or retained introns. This separation facilitates the incorporation of novel methods for splice site detection as well as additional sources of information to yield transcript assemblies that are more accurate. Not only a growing annotation of known splice sites, exon junctions, transcription start and end sites (TSSs and TESs) or even full-length isoforms can guide the assembly for most model organisms, but also additional gene boundary data can aid the interpretation of RNA-seq data. Our experiments demonstrate the superior performance of CIDANE in all these different scenarios of optionally available levels of annotation as well as in the interpretation of additionally available gene boundary data. The general work flow of CIDANE is illustrated in Fig. 1.

## Results and discussion

We compared the performance of CIDANE in reconstructing transcripts from RNA-seq data to existing state-of-the-art methods. We evaluated the prediction quality on the transcript level based on both simulated and real data. While simulated data capture the characteristics of real data only to the extent that we understand the specifics of the experimental protocol, the performance analysis based on real RNA-seq data today still lacks a gold standard RNA-seq library along with annotated expressed transcripts. Therefore, the results of both types of experiments together provide a more meaningful picture of the true performance of a transcript assembly method.

Using simulated data, we investigated the impact of transcript abundance on the prediction quality and considered the scenario where a partial annotation of the (human) transcriptome is available to guide the reconstruction. We assessed both the mere absence or presence of a (true) transcript in the prediction as well as the accuracy of the estimation of their abundances. Generating *perfect mapping files*, we make an attempt to quantify the dependence of current genome-based transcript assembly tools on the accuracy of the read mapping (Additional file 1: Figures S1 and S2). We demonstrate the superiority of CIDANE in the ab initio analysis of two human RNA-seq data sets from the ENCODE project [21], and through an integrated analysis of modENCODE RNA data, including RNA-seq, cap analysis of gene expression (CAGE), and poly(A) site sequencing (PAS-seq), obtained from the heads of 20-day-old adult *Drosophila melanogaster*. CAGE and PAS-seq data facilitate the mapping of TSSs and TESs, which are very difficult to infer from RNA-seq data alone. Furthermore, we illustrate CIDANE’s ability to (i) incorporate prior knowledge to improve substantially the prediction in various realistic scenarios and (ii) recover (*invisible*) transcripts with uncovered splice junctions.

We compared the prediction to a reference transcriptome, referred to as *ground truth*, containing the *true transcripts*. Where not specified otherwise, we consider a true transcript as *recovered* by a predicted transcript if their sequences of introns (intron chains) are identical. A true single-exon transcript is scored as recovered if it overlaps a predicted single-exon transcript. Every predicted transcript is matched to at most one true transcript and vice versa. If rec, true, and pred denote the number of recovered, true, and predicted transcripts, respectively, we applied recall (rec/true), precision (rec/pred), and *F* score, the harmonic mean of recall and precision ((2×precision×recall)/(precision+recall)), as a measure of prediction quality. Not to penalize potential novel discoveries, the calculation of precision ignores predicted transcripts that do not overlap any of the true transcripts. The version number of each tool and the parameters used in our experiments are specified in Additional file 1.

### Isoform reconstruction from simulated data

To obtain data as realistic as possible, we used FluxSimulator [22] to generate RNA-seq data sets based on ∼ 78,000 UCSC-known (February, GRCh37/hg19) human transcripts [23]. After assigning randomized expression levels to all annotated transcripts following a distribution observed in real data, FluxSimulator simulates the individual steps of an RNA-seq experiment, including reverse transcription, fragmentation, size selection, and sequencing. For all simulated data sets used in this section, the parameter files specifying the model of the RNA-seq experiment and the alignments are available from [24].

#### Ab initio transcript assembly

Mimicking the characteristics of real RNA-seq data, we generated four data sets comprising 40 and 80 million read pairs (i.e., 80 and 160 million reads) of length 75 and 100 bp, respectively. The fragment lengths observed after gel electrophoresis are modeled by a normal distribution *N*(250,25) for the 75-bp reads and *N*(300,30) for the 100-bp reads. We mapped each set of paired-end reads to the set of known transcripts using TopHat2 [25]. We defined the ground truth as the set of all annotated transcripts (UCSC) for which at least one paired-end read has been produced.

We compared the performance of CIDANE to the transcriptome reconstruction quality of StringTie [18], Cufflinks [2], CLASS [13], IsoLasso [17], SLIDE [16], and MITIE [11]. All recall and precision values achieved by CIDANE include transcripts with uncovered splice junctions predicted in phase II. We did not include iReckon and GRIT [26] in this first benchmark as both methods require TSSs and TESs to be provided, which, as shown by the experiments in Sections “Annotation-guided assembly” and “Integrating real RNA-seq, CAGE, and PAS-seq”, provides valuable guidance in transcript reconstruction. While IsoLasso, SLIDE, and CIDANE employ known exon–intron boundaries, Cufflinks, CLASS, and StringTie do not allow for the incorporation of pre-computed or annotated splice sites. Cufflinks and StringTie do accept annotated full-length transcripts [27], a scenario that we will investigate in Section “Transcript assembly with partial annotation”. In this experiment, we disable the ability of CIDANE to recombine acceptor and donor sites to form novel exons. Since exon boundary information could be used to infer the originating strand, in the following we apply strand-unspecific evaluation criteria. To eliminate a potential source of inaccuracy prior to the reconstruction algorithm, we provided IsoLasso and SLIDE with the fragment length distribution parameters as estimated by Cufflinks.

**Fig. 2:**
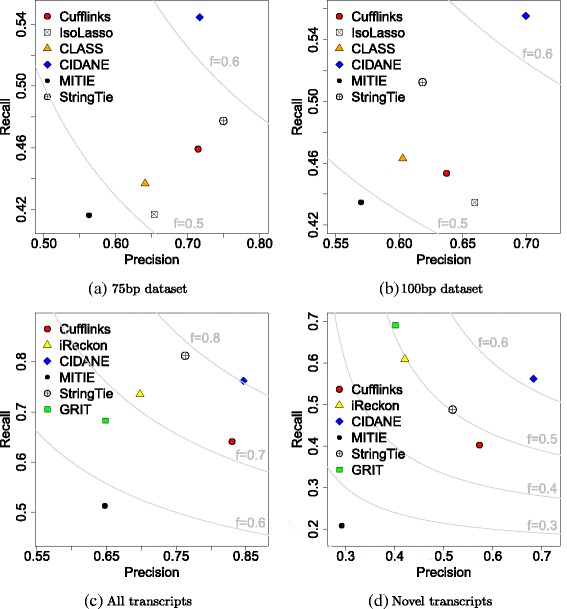
Each tool *X*∈{Cufflinks, IsoLasso, CLASS, CIDANE, MITIE, StringTie } is represented by a point with coordinates (precision of *X*, recall of *X*). *F* score isolines are shown in *light gray*. Simulated data sets comprise 40 million 75-bp (**a**) and 100-bp read pairs (**b**). **c** and **d** Precision and recall achieved by each tool when provided with a partial annotation

For the data set comprising 40 million 75-bp read pairs (Fig. 2a), CIDANE reconstructed transcripts with a recall value of 54.4 *%*, a more than 14 *%* increase over the recall achieved by StringTie (47.7 *%*), Cufflinks (45.9 *%*), or CLASS (43.7 *%*), and a ∼ 30 *%* improvement over IsoLasso (41.7 *%*). At the same time, CIDANE predicts transcripts with a precision like that of Cufflinks (71.6 *%*), and only 4 *%* lower than StringTie (74.9 *%*). When the same number of 100-bp reads is generated (Fig. 2b), the precision of StringTie and Cufflinks decreases significantly and is then lower than CIDANE’s precision by 13.6 *%* and 9.9 *%*, respectively.

IsoLasso seems to suffer from a heuristic determination of the regularization penalty. SLIDE showed the lowest *F* score on all four data sets and was not included in the plots. Note that Cufflinks and CLASS model the transcript reconstruction problem as a *covering* problem minimizing the number of transcripts required to explain the input read alignments qualitatively. Neglecting quantitative information at this stage, it is not surprising that the two methods yield rather conservative predictions. Sections “Transcript assembly with partial annotation”, “Ab initio prediction”, and “Integrating real RNA-seq, CAGE, and PAS-seq” show that the superior performance of CIDANE compared to StringTie and Cufflinks cannot be attributed (only) to the additional exon boundary information. When provided with the exact same partial annotation of transcripts (Section “Transcript assembly with partial annotation”) or when exon boundaries are inferred from the read data alone (Sections “Integrating real RNA-seq, CAGE, and PAS-seq” and “Ab initio prediction”), CIDANE still outperforms all existing methods.

The relative performance of the tools is similar on the larger data sets (Additional file 1: Figure S3). Cufflinks, however, seems to have difficulties assembling the 80 million 100-bp read pairs. Recall and precision achieved by the tools for the four different experimental designs are listed in Additional file 1: Tables S1, S2, S3, and S4.

##### Dependence on transcript abundance

**Fig. 3:**
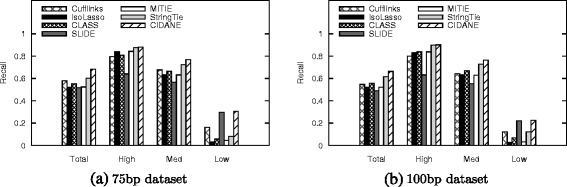
Recall achieved by the different methods vs the expression level of true transcripts in the two 40-million read-pair data sets. Transcripts with simulated FPKM >0.1 (*total*) are grouped into sets *low*, *high*, and *med* that contain the lowest expressed 20 *%* of the transcripts, the highest expressed 5 *%*, and all remaining transcripts, respectively. **a** 75-bp data set. **b** 100-bp data set

Further, we analyzed the influence of transcript abundance on the reconstruction capability of the different methods. We removed all transcripts that have many of their bases uncovered (<0.1 fragments per kilobase of transcript per million fragments sequenced or FPKM) from the ground truth and split the remaining isoforms into three groups: *low* comprises the 20 *%* fraction of transcripts with lowest simulated expression, *high* the highest 5 *%* fraction, and *med* contains the remaining 75 *%* of true transcripts. This subdivision corresponds to cutoffs in relative expression of ∼ 1.5×10^−6^ and ∼ 2.5×10^−4^ molecules, respectively. As expected, a higher abundance facilitates the reconstruction of isoforms (Fig. 3). From the 75-bp reads, however, CIDANE and SLIDE recover almost twice as many lowly expressed isoforms (recall ∼ 31 *%* and ∼ 30 *%*, respectively) as Cufflinks (recall ∼ 6.1 *%*), the next best method, whereas StringTie, CLASS, and IsoLasso recover only ∼ 8 *%*, ∼ 6 *%*, and ∼ 3 *%*, respectively. We observe similar results for the 100-bp data set. Not surprisingly, a higher number of reads facilitates the recovery of low-expressed transcripts (Additional file 1: Figure S4).

The ability of CIDANE to reconstruct, to some extent, even lowly expressed isoforms is likely due to its two core algorithmic improvements: First, CIDANE computes the entire regularization path in phase I (see Section “Model fitting”) to find the right balance between prediction accuracy and sparsity. An objective that is skewed towards sparsity typically yields predictions that miss low-expressed transcripts. Second, our approach considers a wider range of candidate transcripts than existing methods in phase II (Fig. 1). These include isoforms whose low abundance might cause splice junctions to be uncovered by reads rendering them invisible to other approaches. We investigate this effect in Sections “Delayed recovery of transcripts” and “Recovering invisible transcripts”. Note that for the two 40-million read-pair data sets, SLIDE achieves a similar recall on low-expressed isoforms only at the cost of a significantly lower precision and incurs a several orders of magnitude higher computational cost than CIDANE (see Section “Running times”). From the two 80-million read-pair data sets, CIDANE reconstructs low-expressed transcripts with a ∼ 13 *%* to 17 *%* higher recall compared to SLIDE. All expression-level dependent recall values can be found in Additional file 1: Tables S1, S2, S3, and S4.

#### Transcript assembly with partial annotation

We investigated the ability of Cufflinks, using the RABT approach presented in [27], iReckon [15], MITIE, StringTie, GRIT [26], and CIDANE to exploit an existing but incomplete annotation of transcripts. No other assembly tool allowed us to provide annotated transcripts. Such a partial annotation, available for the human transcriptome and many other studied organisms, can provide valuable guidance for the reconstruction of known isoforms, but algorithms must properly balance the preferential prediction of known transcripts and the detection of novel unknown isoforms.

Our algorithmic scheme allows the incorporation of annotated TSSs and TESs during the isoform inference (see Sections “Candidate isoforms” and “Transcription start and end sites”). CIDANE accounts for a higher confidence in annotated vs novel transcripts by adjusting model parameters (see Sections “Model fitting” and “Phase III: fine-tuning and post-processing”).

From 1440 genes on chromosomes 1 and 2 for which between two and eight isoforms have been annotated, we randomly removed, while preserving all exons, at least one and at most 50 *%* of the known isoforms and provided each tool with the remaining ∼ 65 *%* (*Annot*) of the originally ∼ 6300 known transcripts. The hidden ∼ 35 *%* of annotated transcripts (*Novel*) constitutes the reference set (ground truth) in evaluating the ability of each method to infer novel isoforms in the presence of an incomplete annotation. Among the original ∼ 6300 transcripts, FluxSimulator generated 4 million read pairs (75 bp) from a randomly selected subset of 70 *%* expressed transcripts, which were mapped back by TopHat2. All recall and precision values are listed in Additional file 1: Table S5.

Overall, CIDANE achieves the best trade-off between recognizing known and predicting novel transcripts. With respect to the complete set of expressed transcripts (Fig. 2c), CIDANE correctly assembles 20 *%* more transcripts than Cufflinks (77.2 *%* vs 64.1 *%*), combined with a 12 *%* higher precision than StringTie (85.4 *%* vs 76.3 *%*). StringTie’s slightly higher recall (5 *%*) is entirely based on set *Annot* of transcripts known to each tool. StringTie assigns non-zero expression levels to the vast majority of provided transcripts and therefore, not surprisingly achieves a recall of 99 *%* with respect to known transcripts, but only 49 *%* with respect to novel transcripts (Fig. 2d). CIDANE discovers more than 14 *%* and 39 *%* more novel transcripts than StringTie and Cufflinks, respectively, combined with a 34.6 *%* higher precision than StringTie. Only GRIT and iReckon find a greater number of novel transcripts than CIDANE, but at the cost of a very low precision of 40– 42 *%* (vs 70 *%* for CIDANE) and a low sensitivity with respect to known transcripts (68 *%* GRIT vs 89 *%* CIDANE).

#### Abundance estimation accuracy

In addition to evaluating the absence and presence of true transcripts in the prediction, we compared the accuracy of the abundance estimation of CIDANE to existing methods. We restrict this analysis to set *Annot* (see previous section) to reduce the impact of the performance of isoform inference on the measure of abundance estimation quality. For every transcript in set *Annot*, we compared the predicted FPKM to the true FPKM calculated from the number of simulated paired-end reads. True transcripts that were not predicted by a method were considered as reconstructed with zero abundance. To reduce side effects on the abundance estimation due to very short transcripts, we limit the analysis to transcripts of length at least 500 bp (∼ 98.5 *%*).

We observe similar Pearson correlation coefficients between true and predicted abundances for Cufflinks (0.96), GRIT (0.97), iReckon (0.98), and CIDANE (0.99), a slightly lower value of 0.91 for StringTie, and a much lower value of 0.31 for MITIE (see correlation plots in Additional file 1: Figure S5). To obtain a more detailed picture of the abundance estimation accuracy, we evaluated the relative error of predicted transcript abundances. Adopting the definition in [28], the relative error for a transcript *t* with non-zero true abundance $\theta _{t}^{*}$ and predicted abundance *θt*′ is defined as $|\theta _{t}^{*} - \theta _{t}'| / \theta _{t}^{*}$. For $\theta _{t}^{*} = \theta _{t}' = 0$, the relative error is zero and for $\theta _{t}^{'} > \theta _{t}^{*} = 0$, the relative erroris $\infty $.

**Fig. 4:**
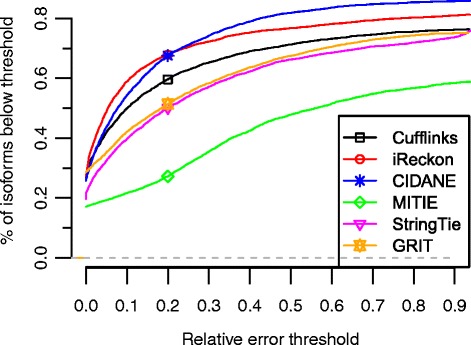
Fraction of correctly predicted expression of known isoforms (*Annot*) at different error thresholds

Figure 4 displays the fraction of annotated transcripts (*Annot*) for which the predicted abundance has a relative error below a certain threshold. Besides iReckon with its very small error rates, CIDANE computes the most accurate estimates of expression levels. By running a tool developed specifically for the statistical estimation of abundances on the set of transcripts assembled by CIDANE, we expect a further improvement in accuracy. A known (or reconstructed) set of expressed transcripts allows for more involved statistical models that often operate, like iReckon’s expectation-maximization algorithm, at single-read resolution.

#### Delayed recovery of transcripts

In this benchmark, we demonstrate the capability of CIDANE to recover in phase II (see Fig. 1) isoforms containing splice junctions that are not supported by any read. Note that a junction between neighboring exons can also be supported (“covered”) by a read pair that maps to the two exons, even if none of the reads span the junction. From the ∼ 6300 transcripts expressed by the genes selected in the previous benchmark set, we simulated 2 million 75-bp read pairs. In all, 118 transcripts had at least one splice junction uncovered and are, therefore, invisible to any method that derives candidate transcripts from a splicing graph representation of the read alignments (see Section “Candidate isoforms”). We note that this simulation neglects sequencing errors and any sequence-specific or positional fragment biases. Furthermore, the mapping of reads to known transcripts is less error-prone than the spliced mapping to a reference genome and, thus, the number of such invisible isoforms is expected to be larger in practice. As before, CIDANE is given only the known exon boundaries and the mapped reads as input. For performance reasons, the delayed generation of transcripts was applied only to genes containing at most 50 exons, covering more than 99 *%* of the genes. For larger genes, CIDANE outputs the initial solution returned by our regularized linear regression approach (phase I in Fig. 1).

CIDANE successfully recovered ∼24.6 *%* of the invisible transcripts expressed in our simulated cellular transcriptome. StringTie, Cufflinks, MITIE, and IsoLasso (provided with exon boundaries) did not predict a single invisible isoform (as expected), while SLIDE recovered ∼ 5 *%*. In rare cases, SLIDE in fact considers candidates with uncovered junctions if otherwise only short candidates with at most two exons exist [29]. We suspect that this strategy is one of the main causes for the very slow running time of SLIDE (see the next section).

When provided with a partial annotation (*Annot*) as in the previous benchmark, iReckon and Cufflinks recovered only one and two isoforms, respectively, whereas CIDANE recovered 17 (∼ 40 *%*) out of 42 invisible transcripts not contained in set *Annot*. StringTie and MITIE again did not predict any invisible transcripts. For each of the three invisible isoforms recovered by iReckon and Cufflinks, the provided annotation (*Annot*) reveals the uncovered splice junction within an alternative isoform. Neither of the methods was able to reconstruct any uncovered novel splice junction.

#### Running times

CIDANE in basic mode (omitting phase II) took 29 min to assemble 80 million read pairs (75 bp), compared to 23 min and 42 min required by StringTie and IsoLasso, respectively. Considering the 2.5 hours TopHat2 took to align the reads, these methods do not constitute the bottleneck of this analysis pipeline. The remaining tools required between 1 hour (Cufflinks) and slightly more than 2 hours (CLASS and MITIE), except for SLIDE, which took more than 62 hours. CIDANE’s optional search for invisible transcripts in phase II requires an additional 42 min of computation. In contrast to methods like StringTie and Cufflinks, the current implementation of CIDANE’s optimization algorithm applied in phase I uses only one thread and can be further enhanced by multi-threading support. The running times of all tools on all five simulated data sets are shown in Additional file 1: Table S6.

### Real human RNA-seq

We illustrate key features of CIDANE on two human RNA-seq data sets from the ENCODE project [21]. Besides the overall performance in terms of recall and precision, we demonstrate CIDANE’s capability to recover transcripts invisible to existing methods and its ability to exploit different levels of annotation to improve the assembly.

The two strand-specific samples obtained from whole B cells in blood (GEO accession GSM981256) and CD14-positive monocytes (GSM984609) comprise 90 million and 120 million 76-bp paired-end reads, respectively, and were aligned using TopHat2. The same data sets were used in [18] to assess StringTie’s performance and we apply, consistently with our other benchmarks, the same evaluation criteria as [18]. We compared transcript predictions to a collection $\mathcal {H}$ of well-curated 171,904 transcripts in 41,409 protein-coding and noncoding genes that was created by the authors in [18] by merging all annotated genes from databases RefSeq [30], Ensembl [31], and the UCSC Browser [32]. Consistent with [18], we included in the reference set (ground truth) all transcripts in $\mathcal {H}$ that had all internal exons and introns covered by (spliced) alignments. As in our other experiments, we considered a (presumably) expressed transcript in the reference set as successfully recovered if the sequence of introns matches perfectly. The precision is defined with respect to all annotated transcripts in $\mathcal {H}$.

#### Ab initio prediction

In our ab initio experiment, only the TopHat2 alignments were provided to CIDANE, StringTie, Cufflinks, IsoLasso, MITIE, and CLASS. SLIDE’s excessive running time did not allow us to include it in the results below. CIDANE’s assembly algorithm was preceded by the algorithm developed in [26] to detect exon boundaries from (spliced) alignments (for more details see Section “Integrating real RNA-seq, CAGE, and PAS-seq”). GRIT and iReckon were not included in this benchmark since they both require TSSs and TESs to be provided.

**Fig. 5:**
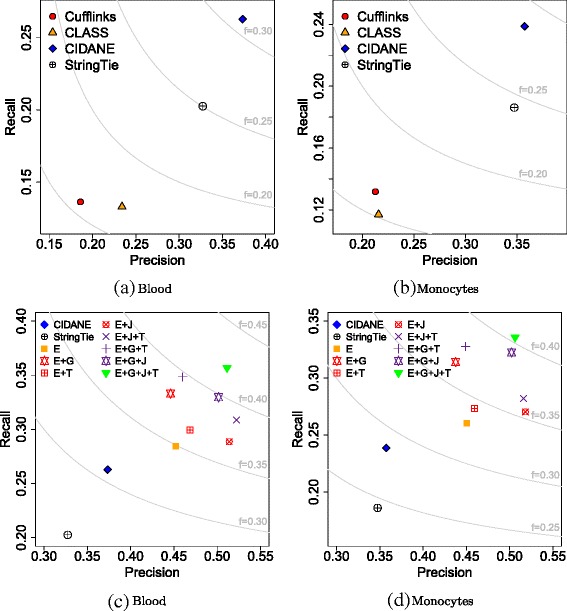
Recall and precision on real human data. **a**, **b** Ab initio transcript predictions. Isolasso and MITIE with recall values of 5–8 % are omitted (see Additional file 1: Table S7). **c**, **d** Recall and precision of annotation-guided assemblies by CIDANE. Ab initio predictions of CIDANE and StringTie shown as reference. In addition to read alignments, CIDANE employs information on exon boundary (*E*), gene boundary (*G*), TSS/TES (*T*), and exon junction (*J*). Same color of symbols indicates same number of augmentary information types

On both data sets, CIDANE reconstructed transcripts with significantly higher sensitivity than all competing methods, while at the same time producing the lowest number of false positive predictions (Fig. 5a, b) and Additional file 1: Table S7). Compared to StringTie and Cufflinks, the two next most sensitive assemblers, CIDANE recovered 28.2 *%* and 81.1 *%* more transcripts expressed in the monocyte sample, and 29.7 *%* and 92.9 *%* more transcripts expressed in the blood sample. CIDANE’s improvement in recall over StringTie translates into an increase of 3412 (14,885 vs 11,473) and 3137 (14,254 vs 11,117) correctly predicted transcripts in the blood and monocyte samples, respectively. The generally low values in sensitivity and precision are due to alignments contributing to the coverage of multiple alternatively spliced isoforms in the definition of our reference set and an incomplete annotation of human transcripts. CIDANE (single-threaded) took 2 and 3.5 hours to assemble the blood and monocyte reads (Additional file 1: Table S6). Only StringTie (using up to 16 threads) was considerably faster. Again, following the analysis pipeline in [18], the preceding alignment of 180 million (blood) and 240 million (monocyte) reads by TopHat2 required 14 and 20 hours, respectively, and constitutes the (computational) bottleneck in this analysis. Nevertheless, multi-threading support will further scale CIDANE’s performance since its core algorithms operate on each locus independently. Cufflinks, for example, achieved a tenfold speedup by using up to 16 threads. Experiments (not shown) on a more sparse formulation of our optimization model (“Methods”, Eq. 1) did improve the running time, but only at the cost of accuracy.

#### Annotation-guided assembly

Any ab initio prediction has to cope with the highly under-determined nature of the RNA-seq puzzle. Depending on the studied species and the specific biological question addressed by the RNA-seq experiment, additional information is often available that can guide the assembly and potentially improve the prediction. As for many well-studied model organisms, well-curated annotations describe the experimentally validated exon–intron structure of human genes. Known splice junctions, for example, are routinely used in the preceding alignment step to facilitate the spliced alignment across introns. Our experiments demonstrate (Fig. 5c, d and Additional file 1: Table S8) that the transcript assembly itself can benefit from such an additional input too. Combined with a small gain in sensitivity, CIDANE’s precision increased by more than 40 *%* and 44 *%* in the blood and monocyte samples, respectively. Even employing exon boundary information alone without the donor–acceptor pairing can reduce the number of false positive transcript predictions considerably.

However, not only an annotation can provide such valuable guidance. Native elongating transcript sequencing (NET-seq), for example, contains an explicit signal on the location of exon boundaries [33]. Strong RNA polymerase II pausing at exon borders manifests in sharp peaks in NET-seq read coverage. Similarly, CAGE and PAS-seq can help to identify TSSs and TESs (see Section “Integrating real RNA-seq, CAGE, and PAS-seq”). Consistent with our observations in Section “Integrating real RNA-seq, CAGE, and PAS-seq”, information available on TSSs and TESs also significantly enhances CIDANE’s assembly of transcripts. If the biological question involves a known set of genes, for example, then gene boundary information prevents fragmentation and fusion of genes caused by missing and ambiguous read alignments, respectively, and thus, helps to improve significantly both the sensitivity and precision of CIDANE’s prediction. Not surprisingly, combining exon boundary, splice junction, gene boundary, or TSS and TES information yields even more accurate transcript reconstructions. At the extreme end of the spectrum, allowing CIDANE to exploit the full information content of the annotated transcriptome yields assemblies of the blood and monocyte reads that contain 10,883 (25,769 vs 14,886) and 14,724 (25,970 vs 14,246) more correctly discovered transcripts than CIDANE’s ab initio prediction, combined with a precision of around 80 *%*. Note that the latter mode of CIDANE does not only estimate the abundance of annotated transcripts. As in Section “Transcript assembly with partial annotation”, CIDANE selects a subset of these transcripts that it believes are expressed and at the same time constructs novel isoforms not yet annotated.

#### Recovering invisible transcripts

In a real data set, it is impossible to distinguish invisible transcripts from transcripts in the curated set $\mathcal {H}$ that are simply not expressed. On the other hand, expressed transcripts that can be correctly identified in a given RNA-seq data set might be invisible in a lower coverage experiment. We, thus, design the experimental evaluation in this section in the reverse way. Starting from a set of (correctly) assembled transcripts $\mathcal {T}$, we uniformly subsample the set of all concordant read-pair alignments (genome-wide) using *samtools*, which renders a subset of transcripts in $\mathcal {T}$ invisible. Additional file 1: Tables S9 and S10 show the number of invisible transcripts for different fractions of sampled reads when the four initial sets of transcripts $\mathcal {T}$ contain all transcripts that were correctly assembled by one of the four most accurate methods based on the full set of 180 million (blood) and 240 million (monocyte) read alignments, respectively. The numbers shown are conservative estimates of the true numbers of invisible transcripts. They do not include transcripts invisible in the full set of alignments and most methods filter the lowest-expressed transcripts for each gene, which would become invisible even if large fractions are sampled. Furthermore, invisible transcripts among false negative predictions are not taken into account either. Nevertheless, even in real RNA-seq data sets containing 54 million (blood) and 72 million (monocyte) reads, between 656 and 949 transcripts that were correctly assembled by StringTie or CIDANE from the full set of reads are invisible (Additional file 1: Tables S9 and S10). More sensitive methods provide a more comprehensive set of transcripts $\mathcal {T}$, yielding a higher number of invisible transcripts $\mathcal {I}$ as reads are removed.

To investigate the utility of CIDANE’s delayed recovery of invisible transcripts, we do not rely on the prediction of any single method, but start from a high-confidence set of transcripts $\mathcal {T}$ that contains all transcripts in the curated set $\mathcal {H}$ that were predicted by both StringTie and CIDANE, the two best-performing methods. As CIDANE and StringTie will agree mostly on highly expressed transcripts, we have to sample randomly fewer read alignments to obtain a reasonable number of invisible transcripts (last columns in Additional file 1: Tables S9 and S10). Among loci that express transcripts with at least one splice junction uncovered by the 54 million (blood) and 72 million (monocyte) read alignments, CIDANE successfully recovers 21.4 *%* and 21.1 *%* of invisible transcripts, at a precision of 34.1 *%* and 31.6 *%*, respectively. CIDANE’s recall is just slightly lower than the one it achieves on the simulated data (see Section “Delayed recovery of transcripts”), despite additional error sources in the real data sets. CIDANE recovers invisible transcripts (phase II) in the blood sample with a precision that is less than 3 percentage points lower than its precision in predicting visible transcripts (phase I) but even higher than the precision achieved by all competing methods on this less-challenging set of transcripts (Additional file 1: Table S7). Transcripts in the monocyte sample generally seem to be more difficult to reconstruct than in the blood sample. Invisible transcripts in the monocyte data set have a lower read coverage than in the blood data set (Additional file 1: Figure S6).

**Fig. 6:**
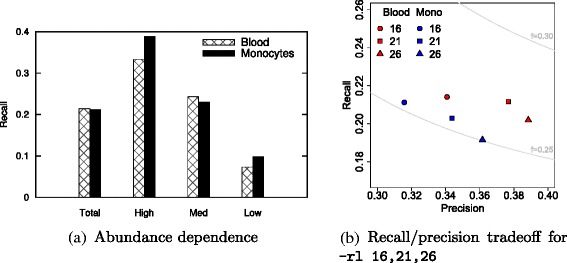
Performance of CIDANE’s delayed detection of invisible transcripts (phase II). **a** Fraction of invisible transcripts recovered among highest expressed 5 *%* (high), lowest expressed 20 *%* (low), and all remaining transcripts (med). **b** Recall/precision trade-off. The precision increases significantly for larger regularization penalties (option -rl), at the cost of a small decrease in recall

In Additional file 1: Tables S11 and S12, we show the results for the recovery of invisible transcripts for samples of size $20~\%, 30~\%, \dots,90~\%$. Overall, CIDANE performs better on larger samples. The smaller the fraction of sampled alignments, the lower the read coverage of invisible transcripts (Additional file 1: Figure S6), which makes their recovery even harder. Similarly, relative expression has an impact on CIDANE’s ability to detect invisible transcripts. Among the 5 *%* highest expressed invisible transcripts in the monocyte sample, CIDANE recovered 39 *%*, while it reconstructed 10 *%* among the 20 *%* lowest expressed invisible transcripts (Fig. 6a). A similar pattern can be observed for different sampling fractions, except for large samples (≥70 *%*) of the monocyte fragments. There, the left tail of the distribution of their expression levels drops less sharply towards transcripts with extremely low read coverage (Additional file 1: Figure S6b), and the small number of invisible transcripts (Additional file 1: Table S10) with estimated reference expression is prone to a higher variance.

Finally, we demonstrate the effect an adjusted regularization penalty in phase II (“Methods”, threshold *λ* in Eq. 4) has on the recall/precision trade-off of CIDANE’s invisible transcript recovery (Fig. 6b). When increasing (in steps of 5 units) the multiplicative factor that controls the cost of transcripts generated in phase II (option -rl), the precision increases to up to 39 *%* (blood) and 36 *%* (monocyte), combined with a decrease in recall by less than 2 percentage points. Again, similar behavior can be observed for other sample sizes (Additional file 1: Tables S11 and S12).

### Integrating real RNA-seq, CAGE, and PAS-seq

RNA-seq data provide an explicit signal for the detection of introns that is more informative than mere read coverage. Spliced alignments span splice junctions between exons and can be leveraged to infer splice donor and splice acceptor sites and thus, the boundary of internal exons. In contrast, the reconstruction of transcript boundaries, i.e., the TSS at the $5^{\prime }$ end and the TES at the $3^{\prime }$ end, relies on a read coverage drop that is blurred by biases in the RNA-seq assay and is thus error-prone.

The conceptual separation of (i) the discovery of exons and (ii) the assembly of exons into transcripts allows CIDANE to employ additional sources of information in both modules. Not only a comprehensive (yet incomplete) annotation available for most model organisms can guide tasks (i) and (ii) (see Sections “Transcript assembly with partial annotation” and “Annotation-guided assembly”), but additional gene boundary data can aid the interpretation of RNA-seq data [26].

By integrating *Drosophila melanogaster* RNA-seq, CAGE, and PAS-seq data, GRIT [26] assembled transcripts with a considerably higher recall and precision than Cufflinks. CAGE and PAS-seq produce reads from the $5^{\prime }$ ends and polyadenylation sites of mRNAs, respectively, and thus facilitate the mapping of TSSs and TESs. Since reconstructing transcripts from RNA-seq data alone is intrinsically underdetermined [34], a mapped TSS/TES can reduce the search space significantly, particularly for complex loci, and this is, thus, expected to yield more accurate transcriptome predictions. In fact, experiments on simulated data performed in [14] suggest the importance of TSS/TES information in transcript assembly.

In this section, we demonstrate the superiority of our comprehensive transcript assembly approach on the integrated analysis of modENCODE RNA data, comprising stranded RNA-seq, CAGE, and PAS-seq data obtained from 20-day-old adult *D. melanogaster* heads [26]. We reconstruct transcripts ab initio without relying on any elements of the annotation of the *D. melanogaster* genome. Instead, we compute exon and transcript boundaries using the boundary discovery procedure of GRIT. Exons and introns are identified by read coverage and spliced alignments, respectively. Gene regions then contain exons that are connected by introns. In addition to splice donor and splice acceptor sites, the TSS and TES are identified from read coverage peaks in the CAGE and PAS-seq data. For details, we refer the interested reader to the original description of the procedure in [26].

Candidate transcripts considered by CIDANE correspond to paths in the splicing graph (see Section “Candidate isoforms”). Only paths from exons whose $5^{\prime }$ boundary coincides with an identified TSS (and ends with a splice donor site) to exons whose $3^{\prime }$ boundary coincides with an identified TES (and begins with a splice acceptor site) are considered. Single-exon transcripts are bounded by an identified TSS and TES on the $5^{\prime }$ and $3^{\prime }$ ends, respectively.

We compared the performance of CIDANE, GRIT (latest version 1.1.2c), StringTie, and Cufflinks on four replicates, two male and two female (see [26] or Additional file 1: Table S13). In the experiments performed in [26] on the same data sets, GRIT drastically outperformed annotation tools Scripture [9] and Trinity+Rsem [35] in terms of recall and precision. Here we apply the same evaluation criteria as in [26] and thus, refrain from benchmarking CIDANE against tools Scripture and Trinity+Rsem. Like [26], we assumed a FlyBase 5.45 [36] transcript to be expressed in our sample if it is composed of a single exon or if otherwise every splice junction is supported by at least one read. Since transcripts contained in the resulting ground truth by definition had no uncovered splice junctions, we disabled the delayed transcript recovery mode (phase II in Fig. 1) of CIDANE. Applying the above criteria, between ∼ 8200 and ∼10,000 transcripts were expressed in each of the four *D. melanogaster* head samples.

We considered an expressed transcript in the resulting ground truth as successfully recovered if the sequence of introns (intron chain) matches perfectly (same criteria as in Sections “Isoform reconstruction from simulated data” and “Real human RNA-seq”) and if optionally the transcript boundaries, i.e., TSS and TES, lie within 50 or 200 bp of each other. The precision is defined with respect to the set of all transcripts annotated in FlyBase.

**Fig. 7:**
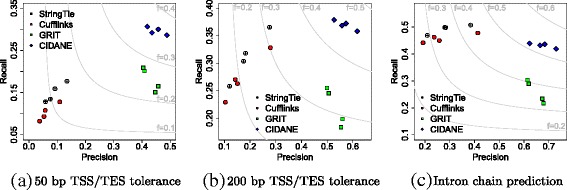
Recall and precision of transcript prediction by Cufflinks, StringTie, GRIT, and CIDANE from integrated RNA data. Different thresholds in TSS/TES accuracy are applied in the true positive definition. **a** 50-bp TSS/TES tolerance. **b** 200-bp TSS/TES tolerance. **c** Intron chain prediction

Figure 7 depicts recall, precision, and *F* score achieved by Cufflinks, StringTie, GRIT, and CIDANE on the identically colored four replicates. Their precise coordinates are listed in Additional file 1: Tables S14, S15, and S16. As was done in [26], we filtered transcripts predicted by GRIT with expression score lower bounds less than 1×10^−6^ estimated FPKM at a marginal 99 *%* significance level.

Figure 7a, b takes into account the accuracy of transcript boundaries with different tolerances. If the predicted and annotated TSS/TES are required to lie within 50 bp of each other (Fig. 7a), the lack of read data on the $5^{\prime }$ ends and polyadenylation sites of mRNAs results in a significantly poorer performance of StringTie and Cufflinks compared to GRIT. Employing the same amount of data as GRIT, however, CIDANE achieves a recall of ∼ 29– 31 *%*, compared to ∼ 15– 21 *%* for GRIT, combined with a slightly higher precision. Utilizing the additional CAGE and PAS-seq data, CIDANE reconstructs transcripts with around threefold to eightfold higher precision than StringTie and fourfold to 12-fold higher precision than Cufflinks. If we relax the TSS/TES tolerance to 200 bp (Fig. 7b), GRIT’s prediction profits from the additional CAGE and PAS-seq data mostly in terms of precision. Again, CIDANE manages to reconstruct substantially more transcripts than GRIT, combined with a slightly higher precision. CIDANE’s gain in precision over StringTie and Cufflinks ranges from about twofold to sixfold.

Figure 7c neglects the accuracy of transcript boundaries. CIDANE (*f*≥0.51) combines the superior precision of GRIT (*f*≥0.33) with the superior recall of Cufflinks (*f*≥0.27) and StringTie (*f*≥0.29) and achieves overall the highest *F* score. Note that the recall values of StringTie and Cufflinks shown in Fig. 7c count annotated transcripts as true positive hits even if there is no evidence for their expression in the CAGE and PAS-seq data. In each analysis, the transcriptome predictions of GRIT and CIDANE are based on the exact same mapping of exons, introns, TSS, and TES. The superiority of our approach results entirely from a more coherent assembly of exons into transcripts.

Concerning the efficiency, CIDANE and StringTie ran for less than 12 and 6 minutes per sample, respectively, while GRIT (allowing up to 16 threads) took ∼ 3 h of computation, including the discovery of exon and transcript boundaries. Cufflinks required slightly more than 1 h of computation per sample.

## Conclusion

We present CIDANE, which provides major improvements in cellular transcriptome reconstruction from RNA-seq over existing assembly tools. Through a carefully chosen trade-off between model complexity and tractability of the resulting optimization problem, and by applying state-of-the-art algorithmic techniques, CIDANE builds full-length transcript models from short sequencing reads with higher recall and precision than was possible before. CIDANE is engineered not only to assemble RNA-seq reads ab initio, but also to make use of the growing annotation of known splice sites, TSSs and TESs, or even full-length transcripts, available for most model organisms. Our experiments show that CIDANE’s core algorithmic engine yields more accurate transcriptome reconstructions than competing tools, in all these different scenarios and under various realistic experimental designs. Along the same lines, CIDANE can employ additional gene boundary data to guide the assembly, thereby improving the precision of the reconstruction significantly.

To some extent, phase II of CIDANE allows us to recover splice junctions that are invisible to all existing approaches. Such junctions are not supported by any read alignment and can be observed predominantly among low-expressed transcripts. While CIDANE in basic mode (phase II omitted) reconstructs a human cellular transcriptome from 80 million aligned read pairs in 29 min, the recovery of invisible junctions is a more complex task. For genes larger than 50 exons, the iterative determination of invisible transcripts might become too expensive in practice and is disabled by default in our current implementation. Future work on the fixed-parameter tractability of the heaviest isoform problem might allow us to push the limits even further.

We expect that CIDANE will provide biologists with accurate transcript predictions from the very large, complex data sets that currently emerge from RNA-seq experiments. Such a high-resolution RNA-seq data interpretation is essential for any type of downstream analysis and will help to expand the catalog of genes and their splice variants.

CIDANE is free open-source software released under the GNU GPL license, and has been developed and tested on a Linux x86_64 system. CIDANE’s source is available from https://bitbucket.org/canzar/cidane.

## Methods

**Fig. 8:**
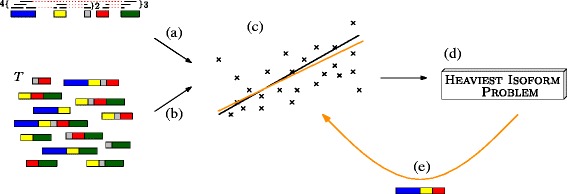
Schematic diagram illustrating CIDANE’s work flow. In the first phase, a linear model is fitted (*black line* in **c**) to a compact representation of the observed read mappings (**a**) using an initial set *T*_init_ of candidate transcripts (**b**). Second, transcripts not in *T*_init_ that can help to improve the prediction are iteratively identified as optimal solutions to the heaviest isoform problem (**d**). The newly constructed isoform (**e**) is used to adjust the fitting (*orange line* in **c**)

In this work, we assume mRNA fragments to be sequenced from both ends, yielding *paired-end reads*. Nonetheless, all results trivially apply to single-end reads. For each locus, identified as connected components of read mappings, CIDANE reconstructs isoforms from RNA-seq data in three phases (Fig. 8). First (Section “Phase I: regularized linear regression”), a linear model is fitted (Fig. 8c) to a compact representation of the observed read mappings (Fig. 8a) using a set of fully supported candidate transcripts (Fig. 8b). Here, our approach differs from existing methods mainly in (i) carefully designed regression coefficients that model (like SLIDE) the distribution of reads along a transcript and in (ii) applying a state-of-the-art machine-learning algorithm to balance the accuracy of the prediction and the number of isoforms assigned a non-zero expression level.

In a second phase (Section “Phase II: delayed generation of improving isoforms”), CIDANE explores the space of transcripts that is neglected by existing methods due to computational challenges. To identify iteratively such a transcript that can help to improve the current prediction, we have to solve a problem (Fig. 8d) that we formalize as the heaviest isoform problem. If the heaviest isoform does not improve the current prediction, CIDANE is guaranteed to have found the best possible set of isoforms without having explicitly enumerated all potential isoforms in the exponentially large space. Otherwise, the newly constructed isoform (Fig. 8e) can be used to adjust our fitting.

Although we show that heaviest isoform problem is NP-complete, we propose an integer linear programming (ILP) formulation that exploits certain properties of RNA-seq data and (optionally) known splicing characteristics that allow for the efficient solution of the ILP. For example, only a few combinations of exons enclosed by two mapped read mates are typically consistent with an estimated fragment length distribution, yielding a small number of variables in our formulation. Furthermore, we (optionally) disregard transcripts whose alternative promoter and polyadenylation sites coincide with acceptor and donor sites of internal exons, since signals read by the transcription and splicing mechanism to identify start (end) sites and acceptor (donor) sites differ significantly. Note that this restriction is conceptually equivalent to considering only *maximal* paths in the splicing graph as candidates, as is done by current methods. CIDANE, however, tries to restore maximal paths that are broken due to uncovered splice junctions. At the same time, the flexibility of an ILP formulation allows CIDANE to incorporate additional data or knowledge concerning, for instance, exon boundaries, intron retentions, TSSs, and TESs.

The prediction is fine-tuned (Section “Phase III: fine-tuning and post-processing”) by refitting the linear model using the initial set of candidate transcripts augmented by all improving transcripts identified in the second phase of CIDANE. Finally, the expression levels of the reconstructed transcripts are re-estimated and converted into FPKM in a post-processing phase (Section “Phase III: fine-tuning and post-processing”).

### Phase I: regularized linear regression

Like count-based methods such as SLIDE and IsoLasso, we summarize the observed read mappings into *segment covers* (Fig. 8a). Instead of trying to explain each read mapping with its precise genomic coordinates, we count the number of reads that fall into non-ambiguously connected segments of the genome. *Segments* in $\mathcal {S}$ represent minimal exon fragments that are covered by reads and bounded by splice sites, TSSs, or TESs (see Additional file 1: Figure S7), derived from spliced alignments, extracted from a set of gene annotations, or supported by additional data. For sequences of segments $\bar {s}_{i}$ and $\bar {s}'_{i}$, a *segment cover*$c_{i}=(\bar {s}_{i},\bar {s}'_{i},b_{i})$ then counts the number *b*_*i*_ of read pairs *r*=(*r*_1_,*r*_2_) where *r*_1_ and *r*_2_ map with a *signature* consistent with $\bar {s}_{i}$ and $\bar {s}'_{i}$, respectively; i.e., the mapping of *r*_1_ (*r*_2_) spans precisely the set of segment boundaries that are implied by $\bar {s}_{i}$ ($\bar {s}'_{i}$) (see Additional file 1: Figure S8). *Faux segment covers*$(\bar {s}_{j},\bar {s}'_{j},0)$ indicate that the corresponding combination of segments was *not* observed in the read data and can help to identify false positive predictions. We denote the set of segment covers, including faux covers (see Additional file 2: Section 1), by $\mathcal {C}$.

#### Candidate isoforms

We derive the initial set of candidate isoforms *T* (Fig. 8b) used to explain the observations (segment covers) as paths in a *splicing graph* [37]. Nodes in a splicing graph correspond to segments $\mathcal {S}$ and edges connect exon fragments whose consecutivity is indicated by (spliced) alignments. Under the assumption that every splice junction of every expressed isoform is covered by at least one mapped read, every expressed (true) transcript is among the paths in the splicing graph. For a formal specification of a splicing graph as employed in CIDANE, see Additional file 2: Section 2.

We further define sets $\mathcal {TSS}$ and $\mathcal {PAS}$, which contain potential TSSs and TESs, respectively. These sets can be compiled from annotated TSSs and polyadenylation sites, additional read data from the $5^{\prime }$ ends and polyadenylation sites of mRNAs (see Section “Integrating real RNA-seq, CAGE, and PAS-seq”), or purely from read mapping data. The latter is based on an exclusion principle. We do not allow for transcripts whose alternative promoter or polyadenylation sites coincide with acceptor and donor sites of internal exons and thus, exclude all segments with spliced alignments supporting their $5^{\prime }$ or $3^{\prime }$ end from $\mathcal {TSS}$ and $\mathcal {PAS}$, respectively. This exclusion strategy is equivalent to considering only *maximal* paths in the graph, as is done by current methods, and can easily be relaxed in CIDANE by setting $\mathcal {TSS}:=\mathcal {S}$ and $\mathcal {PAS}:=\mathcal {S}$.

The set of candidate isoforms among which we select our initial prediction is then obtained by enumerating all (or a preselected set of) paths in the splicing graph that start at a segment in $\mathcal {TSS}$ and end at a segment in $\mathcal {PAS}$.

#### Model fitting

We apply a linear model (Fig. 8c) to estimate the number of reads originating from segments of the genome. Assuming that every position of an expressed transcript is equally likely chosen as a starting position of a sequenced RNA fragment, we model the expected number of fragments mapping to segment cover *c*=(*s*,*s*^′^,*b*) as $\sum _{t\in T} \ell _{t,c} \theta _{t}$, where *ℓ*_*t*,*c*_ is the expected number of starting positions of fragments obtained from transcript *t* that show a mapping signature consistent with *c*. The expression level *θ*_*t*_ of transcript *t* counts the expected number of mapped fragments per transcript base, which is converted to FPKM at a later stage (Section “Phase III: fine-tuning and post-processing”). *ℓ*_*t*,*c*_ depends on the length of segments in $\bar {s}$ and $\bar {s}'$, the length of segments in *t* enclosed by *s* and *s*^′^, the read length, and the cDNA fragment length distribution. Equations defining *ℓ*_*t*,*c*_ as used in our model are given in Additional file 2: Section 3. In contrast, methods like TRAPH [12], MITIE, and IsoInfer/IsoLasso define coefficients *ℓ*_*c*_ that neglect the dependence on transcripts *t*. Note that the distribution of reads along a transcript is generally not uniform, but typically unknown. The same applies to all the experimental data used in this study. Any prior knowledge concerning the likelihood of starting positions can be incorporated into our model through adjusted *ℓ*_*t*,*c*_ coefficients.

We employ the sum of squared errors (i.e., differences between estimated and observed number of reads) as a measure of accuracy of our prediction, weighted by an estimator for the variance of observations *b* [14]. Fitting our model using all candidate transcripts would allow us to fit noise in the data by predicting a large number of isoforms with low but non-zero expression levels. Since in a given cell type really only a small subset of candidate transcripts is expressed, our approach seeks a sparse set of expressed isoforms by augmenting, like SLIDE and IsoLasso, the objective by the *L*^1^ norm of the isoform abundances. Our (initial) prediction *θ*≥0 comprises all transcripts with non-zero expression level in the optimal solution to

(1) $$ \min_{\theta\geq 0} \sum_{\substack{c=(\bar{s},\bar{s}',b): \\ c \in \mathcal{C}}} \left(\frac{b-\sum_{t\in T}{\ell_{t,c}\theta_{t}}}{\sqrt{\max\{\epsilon,b\}}}\right)^{2} + \lambda \sum_{t \in T} \theta_{t}  $$

For faux covers, we replace *b*=0 by *ε* (default *ε*=1). This so-called *Lasso* regression *selects* isoforms by setting the expression levels of all other transcripts to zero one at a time with increasing penalty terms *λ*.

The overall quality of the prediction crucially depends on the right choice of the regularization parameter *λ*. In contrast to previous methods, we balance the relative importance of the accuracy of the prediction and its simplicity (number of transcripts with non-zero expression level) based on the entire path of values for *λ*. As the coefficient path is piecewise linear, the entire regularization path can be computed at the cost of a single least-squares fit [38]. We apply a coordinate descent algorithm implemented in the glmnet Fortran code [39], that cyclically optimizes, for a given *λ*, each isoform abundance separately, holding all other abundances fixed. Update operations (inner products) directly profit from our sparse matrix of *ℓ*_*t*,*c*_ values (see Additional file 2: Section 3). Furthermore, considering a sequence of decreasing values for *λ* exploits estimates at previous *λ*’s as a warm start. After having computed the entire path of values for *λ*, our initial prediction is obtained from the optimal solution to Eq. 1 for the value of *λ* that yields the best *adjustedR*^2^ score. The adjusted *R*^2^ adjusts the goodness of fit (*R*^2^) for the number of isoforms used. If CIDANE is provided with a partial annotation of the transcriptome of an organism, the higher confidence in annotated transcripts is modeled by scaling the regularization penalties *λ* assigned to unknown transcripts by a factor of *γ* (default *γ*=2).

### Phase II: delayed generation of improving isoforms

The aim of the second phase of CIDANE is to recover isoforms with uncovered splice junctions (invisible transcripts) that are not included in the candidate set of the regularized least-squares regression due to their possibly very large number. We employ a delayed column generation technique [20] to identify new candidate isoforms that improve the optimal solution of the regularized least-squares regression without exhaustive enumeration of all possible candidates. Particularly suited for large-scale linear programs, we formulate a piecewise-linear approximation (Additional file 2: Section 4) of the following quadratic program that is equivalent to the regularized least-squares objective function, Eq. 1:

(2) $$\begin{array}{*{20}l} \min&&\sum_{c_{i}\in \mathcal{C}} \left(\frac{e_{i}}{\sqrt{\max\{\epsilon,b_{i}\}}}\right)^{2}& + \lambda \sum_{t \in T} \theta_{t}  \end{array} $$

(3) $$\begin{array}{*{20}l} \text{s.t.}&&\sum_{t\in T}{\ell_{t,c_{i}}\theta_{t}}+ e_{i} &= b_{i}, &&\forall c_{i}\in \mathcal{C} \end{array} $$

$\theta \in \mathbb R^{|T|}_{+}$ is the vector of transcript abundances, and $e\in \mathbb R^{|\mathcal {C}|}$ denotes the vector of errors, i.e., differences between estimated and observed read counts per segment cover. The generation of columns (i.e., variables *θ*_*t*_) is then accomplished by means of an ILP formulation presented below. In the following, we let $m:=|\mathcal {C}|$ be the number of segment covers falling into the considered locus and we let **A** be the corresponding coefficient matrix of constraints, Eq. 3. Since the number of transcripts a gene can potentially encode grows exponentially with the number of its exons, constructing matrix **A** in full is impractical, even for comparatively small genes. Rather, we consider a restricted problem that contains only a small subset of all possible transcripts, represented by the *θ* variables, and generate novel isoforms, i.e., columns of *A*, as needed to improve the overall prediction.

To identify an isoform that can help to improve the prediction in terms of objective Eq. 2, Dantzig’s simplex method [20] requires the determination of a variable (transcript) $\theta _{t_{j}}$ with negative reduced cost $\bar {c}_{j}=\lambda -\mathbf {p}^{T}\mathbf {A}_{j}$, where **p** is the vector of simplex multipliers and **A**_*j*_ is the column of **A** representing transcript *t*_*j*_.

Instead of computing the reduced cost associated with every possible transcript *t*_*j*_, we consider the problem of minimizing $(\lambda -\mathbf {p}^{T}\mathbf {A}_{j})$ over all *t*_*j*_, or equivalently, the problem of maximizing $\mathbf {p}^{T}\mathbf {A}_{j}$ over all transcripts *t*_*j*_. According to constraint Eq. 3, for every 1≤*i*≤*m*, entry *i* of column **A**_*j*_ has value $ \ell _{t_{j},c_{i}}$. The task is, therefore, to find a transcript *t*_*j*_ such that

(4) $$ \sum_{c_{i} \in \mathcal{C}}(p_{i} \ell_{t_{j},c_{i}})>\lambda.  $$

If no such transcript exists, all reduced costs are nonnegative and the current solution is optimal. Next, we model this optimization problem as a variant of the heaviest induced subgraph problem [40] and propose an ILP formulation. For ease of notation, here we only consider the case where reads span single exons. For the general case of reads spanning an arbitrary number of exons, we refer the reader to Additional file 2: Section 5. Consider graph *G*=(*V*,*E*) that contains one vertex for each exon of a locus. We assume that the exons are numbered from left to right from 1 to *n* and identify each vertex by the corresponding exon number. We identify each segment cover $(\bar {s},\bar {s}',b)$ with single-exon sequences $\bar {s}=\langle i \rangle $, $\bar {s}'=\langle j \rangle $ by (*i*,*j*,*b*) and include an edge *e*=(*i*,*j*) in *E*. For each edge *e*∈*E* we denote by $\bar {V}(e)$ the set of vertices whose associated exons lie between the exons given by segments *i* and *j*, i.e., $\bar {V}(e):=\{k\in V: i< k < j\}$. We assign to each edge *e*∈*E* a weight function $w_{e}: \mathcal {P}(\bar {V}(e))\mapsto \mathbb {R}$. Then, finding an improving transcript is equivalent to the following variant of the heaviest induced subgraph problem:

#### **Definition1** (Heaviest isoform problem).

Given graph *G*=(*V*,*E*) and edge weight functions *w*_*e*_, find $T\subseteq V$ such that the induced subgraph has maximal total edge weight, where each induced edge *e* contributes weight $w_{e}(T\cap \bar {V}(e))$.

Edge weights *w*_*e*_ model the corresponding summands on the left-hand side of Eq. 4 and, thus, depend on the selection of exons between the mates of a cover (see Additional file 2: Section 3). In Additional file 2: Section 6, we show that the heaviest isoform problem is NP-complete. For single-end reads that span at most two exons, the weight function is no longer dependent on $T\cap \bar {V}(e)$ and the heaviest isoform problem becomes polynomial-time solvable by a dynamic program.

This problem can be captured by the following integer linear program. For each vertex *i* in *G*, a binary variable *x*_*i*_ indicates whether vertex *i* is contained in the solution. For every edge *e*∈*E* and every set $\bar {V}_{j}\subseteq \bar {V}(e)$, we have a binary variable *y*_*e*,*j*_, which is 1 if and only if vertices selected by the *x* variables are consistent with $\bar {V}_{j}$ and induce *e*, enforced by the constraints below. In the objective function, we let $w_{e,j}:=w_{e}(\bar {V}_{j})$:

$${\fontsize{8.1}{6}\begin{aligned} \max&& \sum_{e\in E}&\sum_{\bar{V}_{j}\subseteq \bar{V}(e)} w_{e, j}y_{e, j}\\ \text{s.t.}&& y_{e,j}&\geq \sum_{v_{i}\in e\cup \bar{V}_{j}}x_{i}+ \sum_{v_{i}\in\bar{V}(e)\setminus \bar{V}_{j}}(1-x_{i}) &&\!\!\!\!\!-|\bar{V}(e)|-1,\\ &&&&& e\in E,\ \bar{V}_{j}\subseteq \bar{V}(e)\\ && y_{e,j}&\leq x_{i}, && e\in E,\ \bar{V}_{j}\subseteq \bar{V}(e),\ v_{i}\in e\cup \bar{V}_{j} \\ && y_{e,j}&\leq 1-x_{i}, &&e\in E,\ \bar{V}_{j}\subseteq \bar{V}(e),\ v_{i}\in\bar{V}(e)\setminus \bar{V}_{j} \end{aligned}} $$

Depending on the quality of the data (determined by, e.g., sequence-specific or positional biases and read mapping accuracy), an isoform that is built by our ILP formulation might improve the prediction with respect to objective Eq. 1 by balancing, for instance, read coverage fluctuations. To prevent fitting noise in the data, we require novel isoforms to explain segment covers *c* that are not supported by any transcript in the initial solution *T*^∗^ returned by the regularized least-squares regression Eq. 1; i.e., ∀*t*∈*T*^∗^:*ℓ*_*t*,*c*_=0. We refer to this set of initially unsupported segment covers as $\tilde {\mathcal {C}} \subseteq \mathcal {C}$. To reduce the impact of spurious read mappings, we require a certain number *k*_*c*_ of read counts to be observed on the set of newly supported segment covers:

(5) $$ \sum_{c_{i} \in \tilde{\mathcal{C}}} b_{i} \sum_{\bar{V}_{j}\subseteq \bar{V}(e_{i})} y_{e_{i},j} \geq k_{c}  $$

Intuitively, variables *y*_*e*,*l*_ associated with an edge *e*=(*i*,*j*) guess the selection of exons between exons *i* and *j*. Since for large *j*−*i* their exponential number would render our ILP approach infeasible, we neglect sets $\bar {V}_{j}$ that would imply fragments of very unlikely length. More precisely, we apply lower and upper bounds $\check {\ell }$ and $\hat {\ell }$ in the computation of *ℓ*_*t*,*c*_ (see Eq. (1) in Additional file 2) that limit the lower and upper 5 *%* quantiles, respectively, of the estimated fragment length distribution. In Additional file 2: Section 7, we translate this fragment length restriction into lower and upper limits on the total length of exons in $\bar {V}_{j}$, which allow us to enumerate feasible exon combinations in $\bar {V}_{j}$ by an efficient splicing-graph-based backtracking scheme.

The construction of improving transcripts can be further guided by additional information such as exon–intron boundaries, TSSs, TESs, or exon connectivity. In the following, we introduce constraints that we optionally add to our ILP formulation, depending on the type of data available, to ensure that the *x* variables encode a transcript that exhibits the desired structure.

#### Exon compatibility

Splice acceptor and splice donor sites can be derived from spliced alignments or extracted from a set of gene annotations. Here we consider the case of a set of known exons $\mathcal {E}$. The more general case where the pairing of alternative acceptor and donor sites is unknown can be reduced to this case by simply including all possible combinations of acceptor and donor sites of an exon in $\mathcal {E}$. Alternatively, the structure of a splicing graph along with the individual mapping of acceptor and donor sites can be enforced through exon connectivity constraints as shown in the next section.

To ensure that the segments in $\mathcal {S}$ selected by the *x* variables form only valid exons in $\mathcal {E}$, we link the segments of each exon $E_{j}\in \mathcal {E}$ by an indicator variable *z*_*j*_:

(6) $$ x_{i} = \sum_{E_{j} \ni s_{i}} z_{j}, \quad 1 \leq i \leq |\mathcal{S}|   $$

This constraint implies that (i) every selected segment *s*_*i*_ (i.e., *x*_*i*_=1) must be part of exactly one selected exon *E*_*j*_ (i.e., *z*_*j*_=1), (ii) all segments of a selected exon must be included, and (iii) no pair of overlapping, and hence incompatible, exons can be selected simultaneously.

#### Exon connectivity

For some complex genes, it is computationally infeasible to enumerate all paths in the splicing graph to obtain the set of candidate isoforms. For such genes, our delayed isoform generation approach allows the exploration of all candidate isoforms without explicitly enumerating them. Constraint Eq. 7 with *u*_*i*,*j*_:=0, therefore, captures the splicing graph structure in a way that the path induced by the selected set of segments agrees with the set of edges *E* in the splicing graph. A simultaneous selection of two segments *s*_*i*_ and *s*_*j*_, *i*<*j*, without selecting any segment *s*_*k*_ with *i*<*k*<*j* is not feasible if the splicing graph does not contain edge (*v*_*i*_,*v*_*j*_). Notice that this scheme allows us to assemble novel exons by selecting acceptor sites (incoming edge) and donor sites (outgoing edge) independently.

Alternatively, we can allow up to *k* (default *k*=2) new edges to be selected from a set of “valuable” edges *E*^′^ missing in the splicing graph. At most *k* binary variables *u*_*i*,*j*_, $1\leq i < j \leq |\mathcal {S}|$, can be set to 1 for (*v*_*i*_,*v*_*j*_)∉*E* to relax the corresponding constraint Eq. 7. We experimented with valuable sets of edges *E*^′^ that allow the explanation of observed covers that cannot be explained solely using edges in *E*. In general, however, any novel intron can be simply modeled by a corresponding edge in *E*^′^:

(7) $${\kern8pt} {\fontsize{9.2pt}{9.6pt}{\begin{aligned} {}1+u_{i,j} &\geq x_{i} + x_{j}- \sum_{i<k<j} x_{k}, &&\!\! 1\leq i < j \leq |\mathcal{S}|,\ (v_{i},v_{j})\notin E  \end{aligned}}}  $$

(8) $$ {}\sum_{(i,j)\in E'} u_{i,j} \leq k   $$

(9) $${\kern8pt} {\fontsize{8.8pt}{9.6pt}{\begin{aligned} u_{i,j} &= 0, && \qquad\qquad\qquad \!\!1\leq i < j \leq |\mathcal{S}|,\ (v_{i},v_{j})\notin E \cup E' \end{aligned}}}  $$

#### Transcription start and end sites

We also have to ensure that improving transcripts built by our ILP start at segments in $\mathcal {TSS}$ and end at segments in $\mathcal {PAS}$. Our model captures both the *exclusion* of potential TSSs and TESs from spliced alignments (see Section “Candidate isoforms”), and the *inclusion* of transcript boundaries, from, e.g., a RNA-seq read coverage drop or from additional reads from the $5^{\prime }$ ends and polyadenylation sites of mRNAs (see Section “Integrating real RNA-seq, CAGE, and PAS-seq”).

Variables *ss*_*i*_ and *es*_*i*_ indicate the start and terminal segment of the generated isoform, respectively. We must select precisely one TSS and one TES (constraints Eqs. 10 and 11) from sets $\mathcal {TSS}$ and $\mathcal {PAS}$, respectively (constraints Eqs. 12 and 13). Designated start and end sites must be part of the predicted transcript (constraints Eqs. 14 and 15). Finally, no segment upstream of the start segment Eq. 16 and no segment downstream of the end segment Eq. 17 can be part of the predicted isoform:

(10) $$\begin{array}{*{20}l} \sum_{v_{i}\in V} \mathit{ss}_{i} &= 1  \end{array} $$

(11) $$\begin{array}{*{20}l}[-2pt] \sum_{v_{i}\in V} \mathit{es}_{i} &= 1  \end{array} $$

(12) $$\begin{array}{*{20}l}[-2pt] \mathit{ss}_{i} &= 0, & v_{i}\notin \mathcal{TSS}  \end{array} $$

(13) $$\begin{array}{*{20}l}[-2pt] \mathit{es}_{i} &= 0, & v_{i}\notin \mathcal{PAS}  \end{array} $$

(14) $$\begin{array}{*{20}l}[-2pt] x_{i} &\geq ss_{i}, & v_{i}\in V  \end{array} $$

(15) $$\begin{array}{*{20}l}[-2pt] x_{i} &\geq \mathit{es}_{i}, & v_{i}\in V  \end{array} $$

(16) $$\begin{array}{*{20}l}[-2pt] x_{i} &\leq 1 - \sum_{j=i+1}^{|V|} ss_{j}, & v_{i}\in V  \end{array} $$

(17) $$\begin{array}{*{20}l}[-2pt] x_{i} &\leq 1 - \sum_{j=1}^{i-1} es_{j}, & v_{i}\in V  \end{array} $$

#### Intron retentions

The explicit exon model described in Section “Exon compatibility” captures intron retentions by simply merging the flanking exons and the retained intron into one virtual exon that is added to set $\mathcal {E}$. Similarly, the more general exon connectivity formulation that is based on individual splice sites rather than assembled exons trivially includes the connectivity of intron retentions.

### Phase III: fine-tuning and post-processing

To adjust the regularization penalty *λ* to the increased set of candidate transcripts implicitly considered by the delayed isoform generation approach and to reduce the effect of the piecewise-linear approximation of the loss function, CIDANE re-solves Eq. 1 with the candidate set *T* containing additionally all transcripts generated in the course of the delayed isoform generation phase. We express a higher confidence in fully supported isoforms by selectively increasing *λ*^′^=*α*·*λ* (default *α*=1.3) for delayed generated transcripts.

Let transcripts $T^{*}=\{t_{1},\dots,t_{m}\}$ with non-zero abundance $\theta ^{*}_{t_{1}},\dots,\theta ^{*}_{t_{m}}$ be returned by the regularized regression Eq. 1 solved in phase I, optionally including the additional isoforms provided by our delayed isoform generation approach (phase II). CIDANE determines the final prediction by post-processing *T*^∗^ as follows. First, to avoid biases introduced by the regularization penalties *λ*, we re-solve Eqs. 2 and 3 for *λ*:=0 using set *T*^∗^ instead of *T* to obtain expression levels $\theta _{t_{i}}'$. Second, we re-estimate the expression levels by computing a final assignment of mapped reads to isoforms that is guided by the relative abundances $\theta _{t_{i}}'$:

$$r(t_{j}) = \sum_{c_{i}\in\mathcal{C}:\ell_{t_{j},c_{i}}>0} \quad b_{i} \cdot \frac{{\ell}_{t_{j},c_{i}} \theta_{t_{j}}'}{\sum \limits_{t_{k} \in T^{*}}{\ell}_{t_{k},c_{i}} \theta_{t_{k}}'}, $$

where *r*(*t*_*j*_) is the number of reads assigned to isoform *t*_*j*_. This assignment of reads to isoforms corrects overestimation or underestimation of the total number of reads within a gene due to non-uniform read mapping coverage. For all isoforms *t*_*j*_∈*T*^∗^ with *r*(*t*_*j*_)≥*α* (default *α*=10), we compute transcript expression levels in FPKM and finally return all isoforms whose predicted expression in FPKM is at least *β* percent (default *β*=10) of the expression of the most abundant transcript for the same gene. When run with a partial annotation of the transcriptome of an organism, we increase the expression threshold *β* to 20 *%* for novel transcripts.

### Ethics approval

Not applicable.

## Additional files

Additional file 1
**Additional figures and tables.** (PDF 578 kb)

Additional file 2
**Algorithmic details.** (PDF 238 kb)
