# Supplementary material for: CIDANE: comprehensive isoform discovery and abundance estimation
Source: Genome Biol. 2016 Jan 30;17:16. doi: 10.1186/s13059-015-0865-0 (PMC4734886; doi:10.1186/s13059-015-0865-0)
Supplement: Additional file 1 — Additional figures and tables. (PDF 578 kb) [file 13059_2015_865_MOESM1_ESM.pdf]

Additional File 1
Additional figures and tables for CIDANE: Comprehensive isoform discovery and abundance estimation

S. Canzar S. Andreotti D. Weese K. Reinert G.W. Klau

Versions and parameters of compared tools and CIDANE In our benchmarks we used Cufflinks (version 2.2.1), IsoLasso (version 2.6.1), CLASS (version 2.0.0), iReckon (version 1.0.8), StringTie (version 1.0.4), SLIDE (latest version, last update May 7th, 2012), MITIE (GitHub version from July 9th, 2014), and GRIT (latest version 1.1.2c). We used the default parameters for each tool except for the following settings: In the benchmark with incomplete annotation Cufflinks was used with option `-g` to provide the annotation and with `--no-effective-length-correction`, since the ground truth FPKM values were computed without effective length correction. IsoLasso was provided with the mean and standard deviation estimated for the fragment length distribution by Cufflinks (option `-p`) and, for the simulation experiment, with all exon boundaries (`-e`). StringTie was run with default parameters and the partial annotation was provided using the `-G` option. For SLIDE we set the parameters of fragment length distribution estimated by Cufflinks directly in the source code as SLIDE cannot read them from command line. Running MITIE involves three programs (define_regions, generate_segment_graph, and transcript_prediction) that were provided with the following non-default parameters, to tune its performance: define_regions (`--cut-regions`), generate_segment_graph (`--few-regions, --region-filter 100`) and transcript_prediction (`--order` 2). For the partial annotation benchmark we further used option (`--C-num-trans 10`) for transcript_prediction. Unless explicitly stated otherwise, CIDANE was run with default parameters. A lower sequencing depth allows (computationally) for a finer approximation of the quadratic objective (see Additional file 1, Section 4). We thus enforce a smaller error bound μ in the two simulated datasets comprising 40 million read pairs using `-u 0.0001`. CIDANE's initial prediction computed in Phase-I fit the simulated data better than can be observed in real data sets (sequencing errors, sequence specific and positional fragment biases). We thus take account of the larger adjusted R^2 scores returned by CIDANE in Phase I and decrease the regularization penalties applied in Phase II in all simulated benchmarks by 10% (`-rl 0.9`).

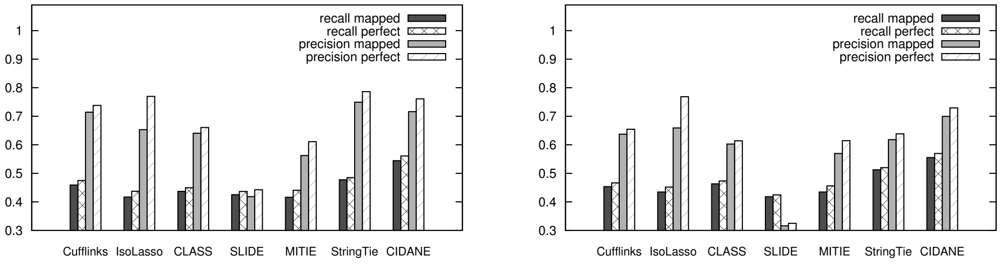

(a) 75bp dataset

(b) 100bp dataset

Figure S1: Dependence on mapping accuracy on both 40 million read pairs datasets. Recall and precision of each tool is shown when provided a perfect BAM file (”perfect”) and when provided the mappings computed by TopHat2 (”mapped”).

Dependence on mapping accuracy In contrast to *de novo* assembly approaches, genome-based methods depend on the accuracy of the preceding mapping of reads to a reference genome. In this Section we make a first attempt to quantify this dependence. From reads simulated by the FluxSimulator, we generated a *perfect BAM file* that mapped each read to its true origin. In contrast, a BAM file output by TopHat2 or any other RNA-seq read aligner will generally contain incorrect mappings of reads caused by sequencing errors, polymorphisms, splicing, and read ambiguity due to repeats. We ran Cufflinks, IsoLasso, CLASS, MITIE, StringTie and CIDANE on the perfect BAM files of all four simulated data sets and compared recall and precision of the transcript assembly to the performance of the tools when provided with the BAM files generated by TopHat2 instead (Figures S1, S2 and Tables S1-S4). The difference in prediction accuracy will be a rather conservative estimate on the mapping dependency, since we expect a larger fraction of reads to be mapped incorrectly in real data than in idealized simulated data that neglect sequencing errors and certain types of biases. Nevertheless, when assembling the 40 million 75bp read pairs we observe a $1.1 - 2.4$ and $2.0 - 11.7$ percentage point improvement in recall and precision, respectively, when the true origin of all reads is known to the assembly tools (Figure 1(a)). Generally, assembly tools seem to benefit mostly in terms of precision rather than recall, independent of the experimental design. IsoLasso's prediction precision seems to depend the most from perfectly aligned reads.

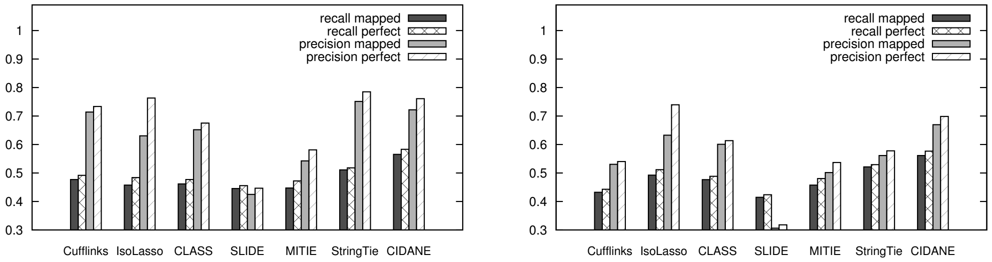

(a) 75bp dataset

(b) 100bp dataset

Figure S2: Dependence on mapping accuracy on both 80 million read pairs datasets. Recall and precision of each tool is shown when provided a perfect BAM file ("perfect") and when provided the mappings computed by TopHat2 ("mapped").

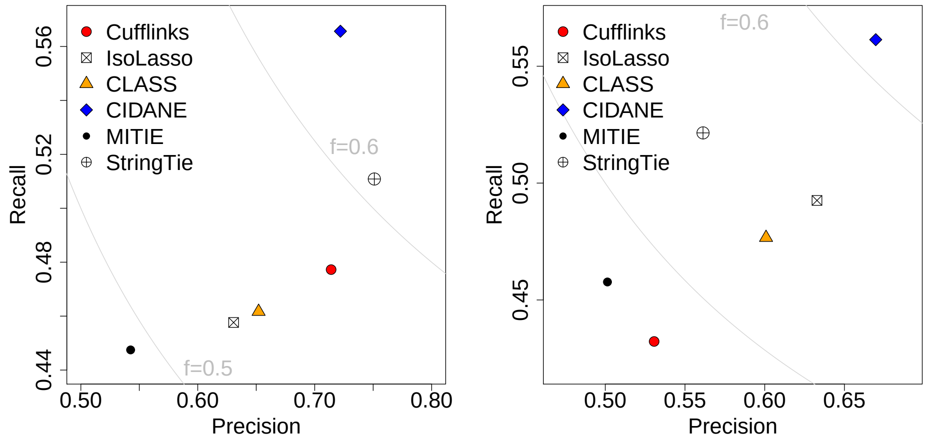

(a) 75bp dataset

(b) 100bp dataset

Figure S3: Each tool $X \in$ {Cufflinks, IsoLasso, CLASS, CIDANE, MITIE, StringTie} is represented by a point with coordinates (precision of X, recall of X). F-score isolines are shown in light-gray. Simulated datasets comprising 80 million 75bp (a) and 100bp read pairs (b), respectively.

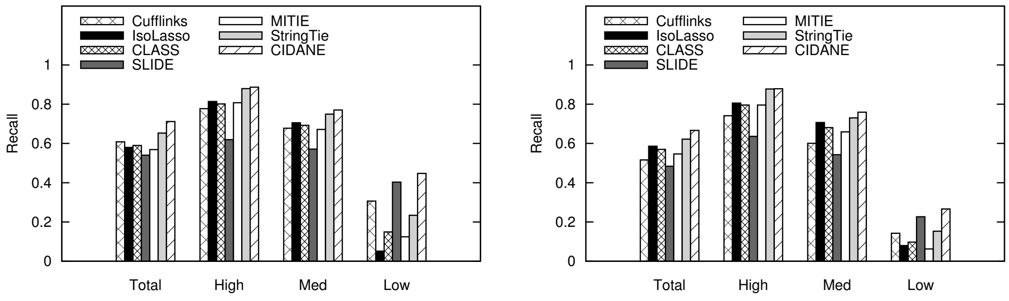

(a) 75bp dataset

(b) 100bp dataset

Figure S4: Recall achieved by the different methods in dependence of the expression level of true transcripts in the two 80 million read pairs datasets. Transcripts with simulated FPKM > 0.1 (*Total*) are grouped into sets *Low*, *High*, and *Med* which contain the lowest expressed 20% of the transcripts, the highest expressed 5%, and all remaining transcripts, respectively.

Table S1: Prediction quality from 40 million read pairs of length 75bp. From left to right, the table shows recall, precision, F-score, recall after removing transcripts with FPKM less than 0.1 from the ground truth, recall of high, medium, and low-expressed transcripts, and recall and precision when providing perfect read mappings.

Tool	recall	precision	F-score	>=0.1	High	Med	Low	recall pf	precision pf
Cufflinks	0.46	0.71	0.56	0.58	0.79	0.68	0.16	0.47	0.74
IsoLasso	0.42	0.65	0.51	0.52	0.84	0.63	0.03	0.44	0.77
CLASS	0.44	0.64	0.52	0.55	0.81	0.67	0.06	0.45	0.66
SLIDE	0.43	0.42	0.42	0.52	0.64	0.57	0.30	0.44	0.44
CIDANE	0.54	0.72	0.62	0.68	0.88	0.77	0.31	0.56	0.76
MITIE	0.42	0.56	0.48	0.52	0.84	0.63	0.04	0.44	0.61
StringTie	0.48	0.75	0.58	0.60	0.88	0.72	0.08	0.49	0.78

Table S2: Prediction quality from 80 million read pairs of length 75bp. From left to right, the table shows recall, precision, F-score, recall after removing transcripts with FPKM less than 0.1 from the ground truth, recall of high, medium, and low-expressed transcripts, and recall and precision when providing perfect read mappings.

Tool	recall	precision	F-score	>=0.1	High	Med	Low	recall pf	precision pf
Cufflinks	0.48	0.71	0.57	0.61	0.78	0.68	0.31	0.49	0.73
IsoLasso	0.46	0.63	0.53	0.58	0.81	0.71	0.05	0.48	0.76
CLASS	0.46	0.65	0.54	0.59	0.80	0.69	0.15	0.48	0.68
SLIDE	0.45	0.42	0.43	0.54	0.62	0.57	0.40	0.46	0.45
CIDANE	0.57	0.72	0.63	0.71	0.89	0.77	0.45	0.58	0.76
MITIE	0.45	0.54	0.49	0.57	0.81	0.67	0.12	0.47	0.58
StringTie	0.51	0.75	0.61	0.65	0.88	0.75	0.23	0.52	0.79

Table S3: Prediction quality from 40 million read pairs of length 100bp. From left to right, the table shows recall, precision, F-score, recall after removing transcripts with FPKM less than 0.1 from the ground truth, recall of high, medium, and low-expressed transcripts, and recall and precision when providing perfect read mappings.

Tool	recall	precision	F-score	>=0.1	High	Med	Low	recall pf	precision pf
Cufflinks	0.45	0.64	0.53	0.55	0.80	0.64	0.12	0.47	0.65
IsoLasso	0.43	0.66	0.52	0.52	0.83	0.63	0.03	0.45	0.77
CLASS	0.46	0.60	0.52	0.56	0.84	0.67	0.07	0.47	0.61
SLIDE	0.42	0.32	0.36	0.49	0.63	0.55	0.22	0.42	0.32
CIDANE	0.56	0.70	0.62	0.66	0.90	0.77	0.22	0.57	0.73
MITIE	0.43	0.57	0.49	0.52	0.84	0.63	0.03	0.46	0.61
StringTie	0.51	0.62	0.56	0.62	0.90	0.73	0.12	0.52	0.64

Table S4: Prediction quality from 80 million read pairs of length 100bp. From left to right, the table shows recall, precision, F-score, recall after removing transcripts with FPKM less than 0.1 from the ground truth, recall of high, medium, and low-expressed transcripts, and recall and precision when providing perfect read mappings.

Tool	recall	precision	F-score	>=0.1	High	Med	Low	recall pf	precision pf
Cufflinks	0.43	0.53	0.48	0.52	0.74	0.60	0.14	0.44	0.54
IsoLasso	0.49	0.63	0.55	0.59	0.81	0.71	0.08	0.51	0.74
CLASS	0.48	0.60	0.53	0.57	0.80	0.68	0.10	0.49	0.61
SLIDE	0.41	0.31	0.35	0.48	0.64	0.54	0.23	0.42	0.32
CIDANE	0.56	0.67	0.61	0.67	0.88	0.76	0.27	0.58	0.70
MITIE	0.46	0.50	0.48	0.55	0.80	0.66	0.06	0.48	0.54
StringTie	0.52	0.56	0.54	0.62	0.88	0.73	0.15	0.53	0.58

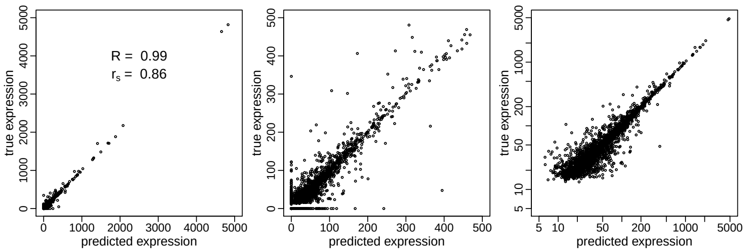

(a) CIDANE

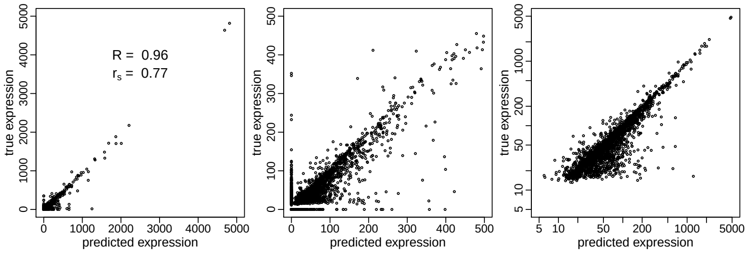

(b) Cufflinks

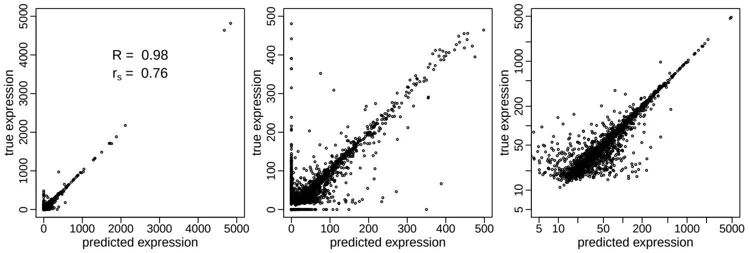

(c) iReckon

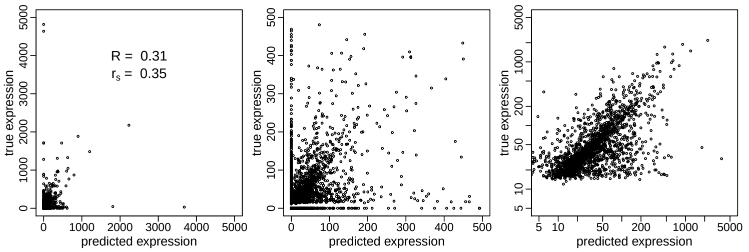

(d) MITIE

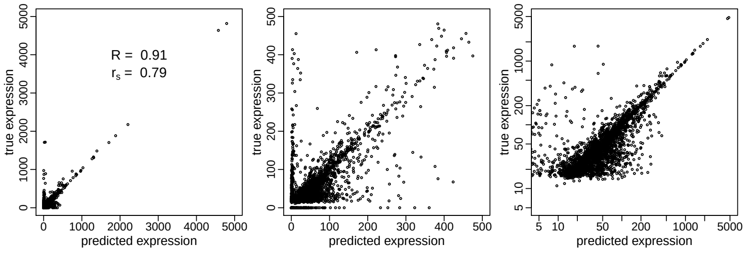

(e) StringTie

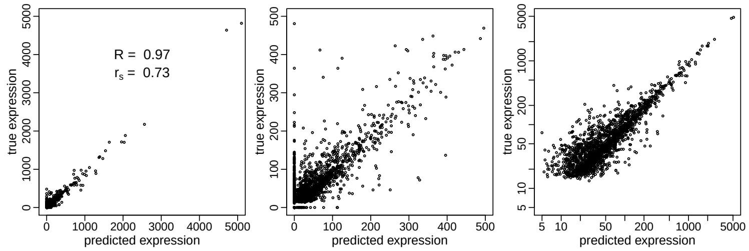

(f) GRIT

Figure S5: Correlation (R:= Pearson correlation coefficient, r_s:= Spearman's rank correlation coefficient) between simulated and predicted transcript abundance in FPKM on the set Annot for (a) CIDANE, (b) Cufflinks, (c) iReckon, (d) MITIE, (e) StringTie, and (f) GRIT. For each tool the left figure plots simulated FPKM value against predicted FPKM value (including predicted FPKM values of 0). The middle figure zooms into FPKM values below 500. The right figure shows the plot of simulated against predicted FPKM values in log-scale after removing transcripts with predicted FPKM value 0.

Table S5: Recall and precision on simulated reads when provided with a partial annotation. Values with respect to the complete set of expressed transcripts (*All*), the set of transcripts contained in the partial annotation (*Annot*), and the expressed transcripts not contained in the partial annotation (*Novel*).

	All		Annot		Novel	
Tool	recall	precision	recall	precision	recall	precision
Cufflinks	0.64	0.83	0.77	0.95	0.40	0.57
iReckon	0.74	0.70	0.80	0.96	0.61	0.42
CIDANE	0.77	0.85	0.89	0.93	0.56	0.70
MITIE	0.51	0.65	0.66	0.79	0.21	0.29
StringTie	0.81	0.76	0.99	0.87	0.49	0.52
GRIT	0.68	0.65	0.68	0.98	0.69	0.40

Table S6: Running times in minutes. All simulation benchmarks were performed on a machine equipped with 2 Intel Xeon CPU X5550 @2.67GHz Quad Core and 72 GB memory, and all tools with multithreading support (Cufflinks, iReckon, SLIDE, MITIE, StringTie) were allowed to use up to 16 threads. The preceding alignment of reads through TopHat2 took 14 hour for the blood sample, and 20 hour for the monocytes sample.

	Simulated data					Encode data	
	(75,40)	(75,80)	(100,40)	(100,80)	(75,4)	Blood	Monocytes
CIDANE (basic)	15	29	30	39	1.1	113	211
CIDANE (full)	44	71	68	66	N/A	N/A	N/A
Cufflinks	25	59	28	52	4.0	273	337
StringTie	8.3	23	13	23	0.7	12	15
IsoLasso	28	42	20	40	N/A	123	137
MITIE	37	123	50	71	5.5	3692	2695
SLIDE	3336	3737	5727	6057	N/A	N/A	N/A
CLASS	72	132	89	190	N/A	101	125
iReckon	N/A	N/A	N/A	N/A	186	N/A	N/A
GRIT	N/A	N/A	N/A	N/A	26	N/A	N/A

Table S7: Recall and precision values on real datasets for blood and monocytes.

monocytes			blood		
Tool	recall	precision	Tool	recall	precision
Cufflinks	0.13	0.21	Cufflinks	0.14	0.19
IsoLasso	0.06	0.17	IsoLasso	0.07	0.19
MITIE	0.08	0.25	MITIE	0.08	0.25
CLASS	0.12	0.22	CLASS	0.13	0.23
StringTie	0.19	0.35	StringTie	0.20	0.33
CIDANE	0.24	0.36	CIDANE	0.26	0.37

Table S8: Recall and precision of CIDANE on real RNA-Seq data for different levels of annotation.

Tool/Annotation	monocytes		blood	
	recall	precision	recall	precision
CIDANE	0.24	0.36	0.26	0.37
StringTie	0.19	0.35	0.20	0.33
exon boundary (E)	0.26	0.45	0.28	0.45
E + gene boundary (E+G)	0.31	0.44	0.33	0.45
E + tss/tes (E+T)	0.27	0.46	0.30	0.47
E + splice junctions (E+J)	0.27	0.52	0.29	0.51
E+J+T	0.28	0.52	0.31	0.52
E+G+T	0.33	0.45	0.35	0.46
E+G+J	0.32	0.50	0.33	0.50
E+G+J+T	0.34	0.51	0.36	0.51
annotated transcripts	0.43	0.80	0.46	0.81

Table S9: Different fractions x of the 180 million reads in the blood data set are sampled, using `samtools view -s x`. For each sample size we give the number of reads (#reads) and the number of invisible transcripts among reliable transcript assembled (from 100% reads) by Cufflinks (Inv Cuff), StringTie (Inv ST), IsoLasso (Inv IL), and CIDANE (Inv CID). The last column gives the number of invisible transcripts in the intersection of true positives of StringTie and CIDANE.)

fraction	#reads	Inv Cuff	Inv ST	Inv IL	Inv CID	Inv CID∩ST
20%	36	695	1304	450	1640	772
30%	54	419	775	256	949	411
40%	72	252	476	143	613	242
50%	90	149	278	74	391	148
60%	108	111	212	53	282	107
70%	126	69	123	26	176	63
80%	144	44	72	14	111	40
90%	162	18	30	5	46	16

Table S10: Different fractions x of the 240 million reads in the monocytes data set are sampled, using `samtools view -s x`. For each sample size we give the number of reads (#reads) and the number of invisible transcripts among reliable transcript assembled (from 100% reads) by Cufflinks (Inv Cuff), StringTie (Inv ST), IsoLasso (Inv IL), and CIDANE (Inv CID). The last column gives the number of invisible transcripts in the intersection of true positives of StringTie and CIDANE.)

fraction	#reads	Inv Cuff	Inv ST	Inv IL	Inv CID	Inv CID∩ST
20%	48	706	1089	374	1570	624
30%	72	430	656	230	946	355
40%	96	268	408	129	604	207
50%	120	169	278	79	408	135
60%	144	112	177	46	263	82
70%	168	64	97	24	146	42
80%	192	32	55	13	71	24
90%	216	12	24	8	26	9

Table S11: Recall and precision of CIDANE's delayed recovery of invisible transcripts when sampling between 20% and 90% of the 180 million reads in the blood data set. We show overall performance and the recall with respect to highest expressed 5% (rec_high), lowest expressed 20% (rec_low), and remaining transcripts (rec_med). Recall/precision are shown when increasing option `-rl` in steps of 5, starting from 16 (default). This parameter controls the cost of transcripts generated in Phase II.

	Fraction sampled							
	20%	30%	40%	50%	60%	70%	80%	90%
	overall performance							
recall	0.19	0.21	0.21	0.27	0.24	0.32	0.33	0.25
precision	0.34	0.34	0.36	0.40	0.41	0.48	0.49	0.36
	abundance dependent performance							
rec_high	0.26	0.33	0.31	0.75	0.83	0.25	0.50	1.00
rec_med	0.22	0.24	0.23	0.26	0.21	0.32	0.30	0.08
rec_low	0.06	0.07	0.10	0.17	0.19	0.33	0.38	0.67
	recall/precision tradeoff							
rec_21	0.19	0.21	0.20	0.26	0.22	0.27	0.30	0.31
prec_21	0.37	0.38	0.36	0.42	0.43	0.53	0.52	0.46
rec_rl26	0.17	0.20	0.19	0.22	0.19	0.22	0.20	0.13
prec_rl26	0.38	0.39	0.41	0.44	0.47	0.53	0.52	0.38

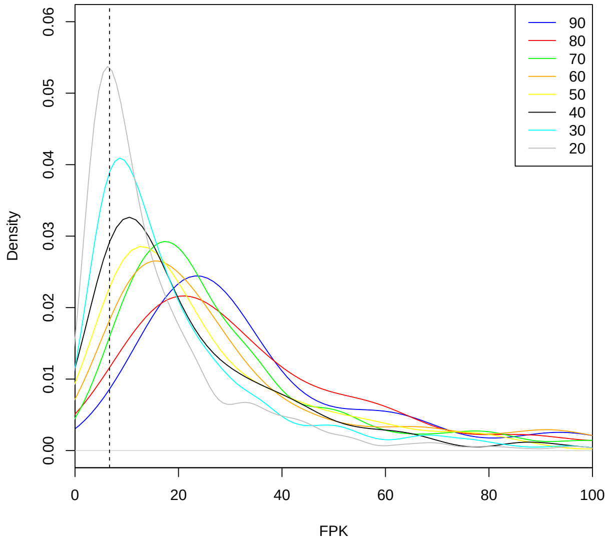

(a) Blood data set

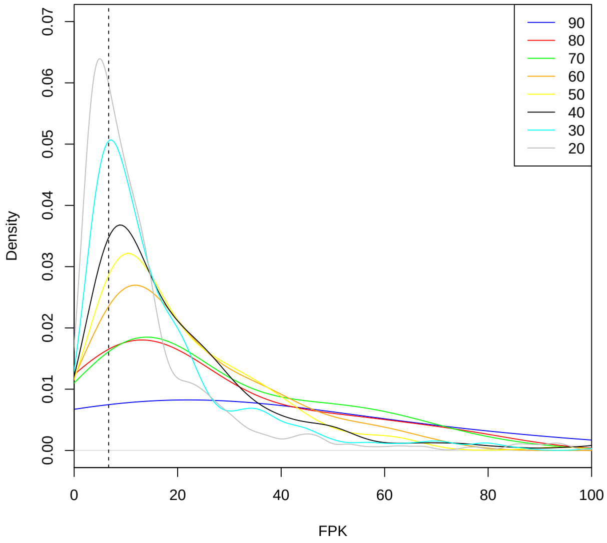

(b) Monocytes data set

Figure S6: Relative number of invisible transcripts with an expression in Fragments per Kilobase of Exon (FPK), for 8 different sampling fractions. Transcripts with relative expressions that lie left of the vertical dashed line (at 6.67) have their bases covered by less than 1 read on average.

Table S12: Recall and precision of CIDANE's delayed recovery of invisible transcripts when sampling between 20% and 90% of the 240 million reads in the monocytes data set. We show overall performance and the recall with respect to highest expressed 5% (rec_high), lowest expressed 20% (rec_low), and remaining transcripts (rec_med). Recall/precision are shown when increasing option `-rl` in steps of 5, starting from 16 (default). This parameter controls the cost of transcripts generated in Phase II.

	Fraction sampled							
	20%	30%	40%	50%	60%	70%	80%	90%
	overall performance							
recall	0.16	0.21	0.21	0.23	0.26	0.21	0.13	0.11
precision	0.27	0.32	0.33	0.33	0.31	0.29	0.25	0.20
	abundance dependent performance							
rec_high	0.31	0.39	0.36	0.00	0.20	0.00	0.00	0.00
rec_med	0.16	0.23	0.23	0.29	0.26	0.26	0.22	0.14
rec_low	0.04	0.10	0.07	0.07	0.13	0.00	0.00	0.00
	recall/precision tradeoff							
rec_rl21	0.15	0.20	0.19	0.21	0.23	0.19	0.17	0.11
prec_rl21	0.28	0.34	0.33	0.34	0.31	0.27	0.27	0.25
rec_rl26	0.14	0.19	0.17	0.21	0.23	0.17	0.17	0.11
prec_rl26	0.30	0.36	0.36	0.35	0.33	0.29	0.31	0.33

Table S13: SRA accession numbers of adult mated 20 days post-eclosion heads

Library Type	Sample ID	Sample Type	SRA Accession Number
CAGE	287	Female	SRR488279
CAGE	290	Male	SRR488280
RNA-seq	287	Female	SRR070420
RNA-seq	288	Female	SRR111882
RNA-seq	290	Male	SRR070421
RNA-seq	291	Male	SRR070424
PAS-seq	288	Female	SRR1151373
PAS-seq	291	Male	SRR1151374

Table S14: Recall and precison of transcript prediction with 50bp TSS/TES tolerance from integrated RNA data. The four *D. melanogaster* samples are specified in TableS13.

	Sample 287		Sample 288		Sample 290		Sample 291	
Tool	recall	precision	recall	precision	recall	precision	recall	precision
Cufflinks	0.08	0.04	0.13	0.11	0.09	0.05	0.11	0.06
GRIT	0.15	0.45	0.17	0.46	0.20	0.41	0.21	0.40
CIDANE	0.29	0.53	0.31	0.42	0.29	0.44	0.30	0.46
StringTie	0.13	0.06	0.18	0.13	0.13	0.08	0.16	0.09

Table S15: Recall and precison of transcript prediction with 200bp TSS/TES tolerance from integrated RNA data. The four *D. melanogaster* samples are specified in TableS13.

	Sample 287		Sample 288		Sample 290		Sample 291	
Tool	recall	precision	recall	precision	recall	precision	recall	precision
Cufflinks	0.23	0.10	0.33	0.28	0.26	0.15	0.27	0.14
GRIT	0.18	0.55	0.20	0.56	0.25	0.51	0.25	0.50
CIDANE	0.36	0.62	0.38	0.53	0.37	0.56	0.37	0.57
StringTie	0.26	0.12	0.37	0.28	0.30	0.17	0.32	0.18

Table S16: Recall and precison of transcript prediction ignoring TSS/TES accuracy from integrated RNA data. The four *D. melanogaster* samples are specified in TableS13.

	Sample 287		Sample 288		Sample 290		Sample 291	
Tool	recall	precision	recall	precision	recall	precision	recall	precision
Cufflinks	0.44	0.19	0.48	0.41	0.45	0.26	0.46	0.24
GRIT	0.22	0.68	0.23	0.67	0.29	0.62	0.30	0.61
CIDANE	0.42	0.73	0.44	0.62	0.43	0.66	0.44	0.68
StringTie	0.47	0.21	0.51	0.38	0.50	0.28	0.50	0.24

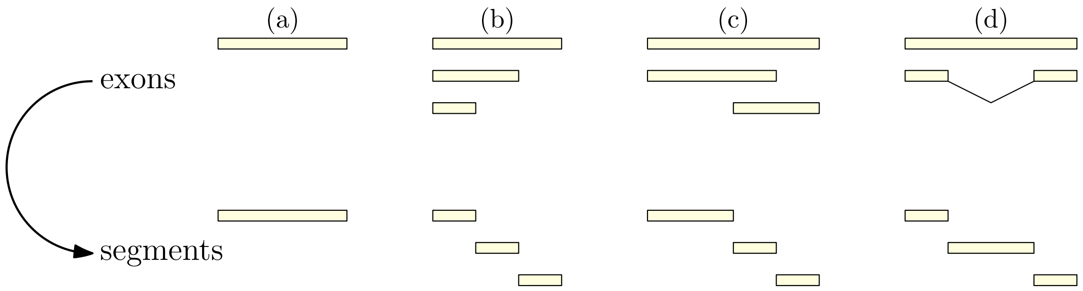

Figure S7: Examples of exons and generated segments. Exons and segments are aligned according to their genomic position. (a) Only a single segment is generated. (b) Top exon has two variants with alternative 3' boundaries, resulting in three segments. (c) Top exon has one variant with alternative 3' boundary and one variant with alternative 5' boundary, also resulting in three segments. (d) Three segments are generated due to an intron retention.

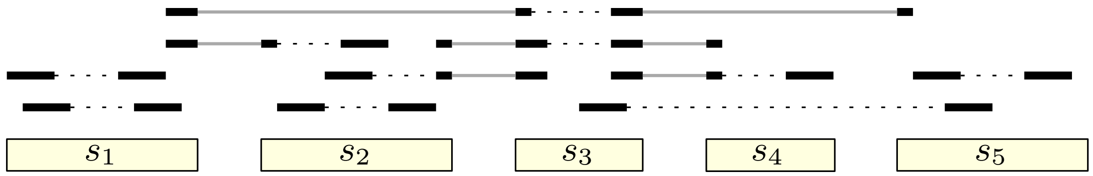

Figure S8: Example of paired-end reads mapped onto segments. Solid lines denote splice junction spanning reads and dotted lines connect paired reads originating from the same fragment. The associated segment covers are given by: $(\langle s_1\rangle, \langle s_1\rangle, 2)$, $(\langle s_1, s_2\rangle, \langle s_2\rangle, 1)$, $(\langle s_1, s_3\rangle, \langle s_3, s_5\rangle, 1)$, $(\langle s_2\rangle, \langle s_2\rangle, 1)$, $(\langle s_2\rangle, \langle s_2, s_3\rangle, 1)$, $(\langle s_2, s_3\rangle, \langle s_3, s_4\rangle, 1)$, $(\langle s_3, s_4\rangle, \langle s_4\rangle), 1)$, $(\langle s_3\rangle, \langle s_5\rangle, 1)$, and $(\langle s_5\rangle, \langle s_5\rangle, 1)$. The segment covers imply an alternative splicing event with skipping of segment s_2.
