# Supplementary material for: CIDANE: comprehensive isoform discovery and abundance estimation
Source: Genome Biol. 2016 Jan 30;17:16. doi: 10.1186/s13059-015-0865-0 (PMC4734886; doi:10.1186/s13059-015-0865-0)
Supplement: Additional file 2 — Algorithmic details. (PDF 238 kb) [file 13059_2015_865_MOESM2_ESM.pdf]

Additional File 2
Algorithmic details for
CIDANE: Comprehensive isoform discovery and abundance estimation

S. Canzar S. Andreotti D. Weese K. Reinert G.W. Klau

1 Faux segment covers

In addition to segment covers counting observed read mappings, our model comprises *faux segment covers* which correspond to combinations of segments not observed in the read data. Faux segment covers provide valuable information that can help to discriminate between true expressed isoforms and false positives. In our current implementation, we add a faux segment cover $(\bar{s}_i, \bar{s}'_i, 0)$ if the following three conditions are satisfied:

1. $\forall (\bar{s}_j, \bar{s}'_j, b_j) \in \mathcal{C} : \bar{s}_j \neq \bar{s}_i \vee \bar{s}'_j \neq \bar{s}'_i$.
2. $|\bar{s}_i| \leq 2 \wedge |\bar{s}'_i| \leq 2$.
3. The splicing graph (see next Section) contains a path from the first segment of $\bar{s}_i$ to the last segment of $\bar{s}'_i$.

2 Splicing graph

A splicing graph [1] represents connectivity properties (edges) of genomic intervals (nodes) inferred from aligned reads. For a given gene or locus let segments in $\mathcal{S}$ be numbered from 1 to $|\mathcal{S}|$ and ordered by their genomic position in $5' \to 3'$ direction. We construct a splicing graph $\mathcal{G}^S = (V, E)$ by adding a vertex $v_i \in V$ for every segment $s_i \in \mathcal{S}$ and two additional start and end vertices v^a and v^b. Let $first(\bar{s})$ and $last(\bar{s})$ denote the first and last segment in the sequence of segments $\bar{s}$, respectively, and let $\preceq$ denote the substring relation. Further, we denote a sequence of segments by "$\langle \ldots \rangle$". Given the set of segment covers $\mathcal{C}$, CIDANE adds a directed edge (v_i, v_j) to E if $i < j$ and one of the following holds:

(i) $s_i = [p_i \mathrel{..} q_i], s_j = [p_j \mathrel{..} q_j]$ and $p_j - q_i = 1$.

(ii) $\exists (\bar{s}, \bar{s}', b) \in \mathcal{C} : \lceil b \rceil \geq \gamma \wedge (\langle s_i, s_j \rangle \preceq \bar{s} \vee \langle s_i, s_j \rangle \preceq \bar{s}')$.

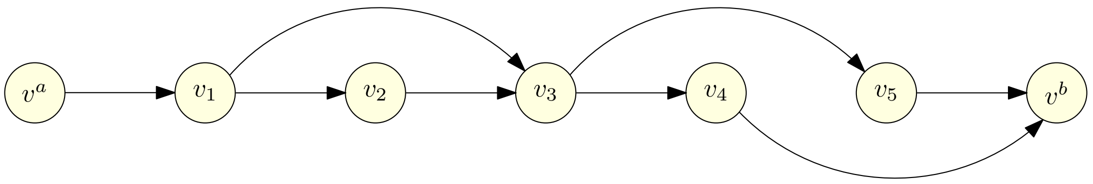

Figure S1: Splicing graph generated for the segment covers shown in Figure S8 in Additional file 2. The graph encodes four transcripts: $t_1 = \langle s_1, s_2, s_3, s_4 \rangle$, $t_2 = \langle s_1, s_2, s_3, s_5 \rangle$, $t_3 = \langle s_1, s_3, s_4 \rangle$ and $t_4 = \langle s_1, s_3, s_5 \rangle$.

(iii) $j = i + 1 \wedge \exists (\bar{s}, \bar{s}', b) \in \mathcal{C} : \lceil b \rceil \geq \gamma \wedge s_i = last(\bar{s}) \wedge s_j = first(\bar{s}')$.

where threshold parameter γ (default: $\gamma = 1$) denotes a lower bound on the number of mapped reads required to support a splice junction. Set E contains an edge between neighboring segments that are not separated by an intron (condition (i)). Condition (ii) implies an edge if a sufficient number of reads was mapped across the associated splice junction. According to (iii), consecutive segments s_i and s_{i+1} are connected by an edge if the associated splice junction is induced by enclosing paired-end reads.

Since candidate transcripts must start at a transcription start site and end at a transcription end site, we add an edge from v^a to all segments in $\mathcal{TSS}$ and an edge from every segment in $\mathcal{TES}$ to v^b. Figure S1 depicts an example splicing graph for the segment covers shown in Figure S8 in Additional file 2. Candidate transcripts considered by CIDANE in Phase I then correspond to all paths from v^a to v^b in $\mathcal{G}^S$.

3 Adjusted segment cover length $\ell_{t,c}$

Given a transcript t, segment cover $c = (\bar{s}, \bar{s}', b)$, and fragment length f, we define $\ell_{t,c}(f)$ as the number of possible starting positions of fragments of length f obtained from transcript t that show a mapping signature consistent with c. If t does not contain $\bar{s}$ or $\bar{s}'$ as substrings, then $\ell_{t,c}(f) = 0$. Otherwise, we compute $\ell_{t,c}(f)$ in two steps: First, for a single read r of length h we determine the leftmost and rightmost starting positions, *lpos* and *rpos*, of r in the transcript sequence of t such that r maps consistent with $\bar{s}$ (analog for $\bar{s}'$). Let s_l and s_k be the first and last segment in $\bar{s}$, respectively. Further, let s_l and s_k span positions $[l_1 \,..\, l_2]$ and $[k_1 \,..\, k_2]$ in the transcript sequence of t, respectively. Then we can compute *lpos* and *rpos* as follows (see also Figure S2):

$$\begin{aligned} lpos &= \max\{l_1, k_1 - h + 1\}\,, \\ rpos &= \min\{l_2, k_2 - h + 1\}\,. \end{aligned}$$

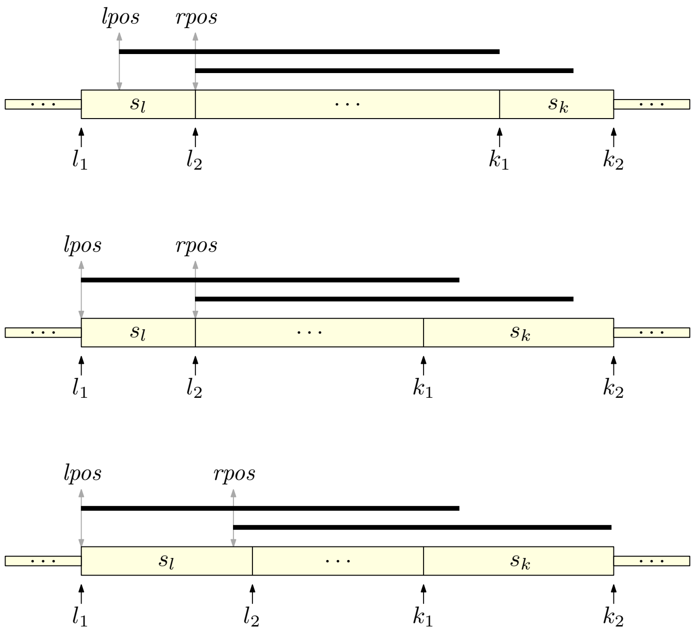

Figure S2: Illustration of values *lpos* and *rpos* for a given transcript comprising a sequence of segments $\langle s_l, \ldots, s_k \rangle$ with segments s_l and s_k spanning positions $[l_1 \mathinner{.\,.} l_2]$ and $[k_1 \mathinner{.\,.} k_2]$, respectively.

From leftmost and rightmost starting positions $(lpos_1, rpos_1)$ for $\bar{s}$ and $(lpos_2, rpos_2)$ for $\bar{s}'$ we can now infer $\ell_{t,c}(f)$: After defining Δ^{in} and Δ^{out} (see Figure S3) as:

$$\Delta^{in} := lpos_2 - rpos_1 \quad \text{and} \quad \Delta^{out} := rpos_2 - lpos_1,$$

we finally obtain

$$\ell_{t,c}(f) = \max\{0, \min\{rpos_1 - lpos_1,\ rpos_2 - lpos_2,\ f - h - \Delta^{in},\ \Delta^{out} + h - f\} + 1\}\,.$$

The length of cDNA fragments, however, is not uniform but typically modeled by a Normal distribution or as truncated Exponential [2]. The current implementation of CIDANE models the fragment length by a Normal distribution with density D, but can easily incorporate alternative fragment length distributions. Then, for a segment cover c and transcript t we compute the *adjusted segment cover length* $\ell_{t,c}$ as follows:

$$\ell_{t,c} := \sum_{f=\check{\ell}}^{\hat{\ell}} D(f)\ell_{t,c}(f)\,, \tag{1}$$

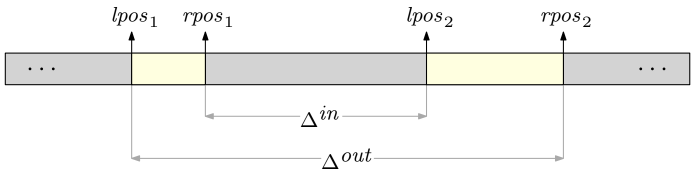

Figure S3: Illustration of values Δ^{in} and Δ^{out} for leftmost and rightmost starting positions $(lpos_1, rpos_1)$ and $(lpos_2, rpos_2)$.

where $\check{\ell}$ and $\hat{\ell}$ are the minimum and maximum considered fragment lengths, respectively, which limit the lower and upper 5%-quantile, respectively, of the estimated fragment length distribution.

4 Piecewise linear approximation of least squares

We approximate the quadratic objective (2) given in the main text by a piecwise linear function as follows. For every $c_i \in \mathcal{C}$ we approximate $g(x) = x^2$ at supporting points $p^i_j \in \mathbb{R}$, $j = 1, \ldots, k$, by its tangent line:

$$g^{p^i_j}(x) = g(p^i_j) + g'(p^i_j)(x - p^i_j) = {p^i_j}^2 + 2p^i_j(x - p^i_j)\,.$$

Due to its convexity, each of $g^{p^i_1}(x), \ldots, g^{p^i_k}(x)$ constitutes a lower bound on $g(x)$. We select the tightest lower bound $\tilde{e}_i \geq \max_{1 \leq j \leq k} g^{p^i_j}(e_i)$, which yields the linear program formulation:

$$\begin{array}{lll}
\min & \displaystyle\sum_{c_i \in \mathcal{C}} \frac{\tilde{e}_i}{\max\{\epsilon, b_i\}} + \lambda \sum_{t \in T} \theta_t & \\
\text{s.t.} & \displaystyle\sum_{t \in T} \ell_{t,c_i}\theta_t + e_i = b_i & \forall c_i \in \mathcal{C} \\
& 2p^i_j e_i - \tilde{e}_i \leq (p^i_j)^2 & 1 \leq i \leq |\mathcal{C}|, 1 \leq j \leq k \\
& \theta_t \in \mathbb{R}_+ & \forall t \in T \\
& e_i \in \mathbb{R} & \forall c_i \in \mathcal{C} \\
& \tilde{e}_i \in \mathbb{R}_+ & \forall c_i \in \mathcal{C}
\end{array}$$

The approximation error of $g^{p^i_j}(x)$ is given by $(x - p^i_j)^2$. Thus, if we want to bound the error of approximating $(e_i/\sqrt{\max\{\epsilon, b_i\}})^2$ to $\mu > 0$, it must hold for $j = 2, \ldots, k$ that $|p^i_j - p^i_{j-1}| \leq 2\sqrt{\mu \max\{\epsilon, b_i\}}$. In a predefined interval $[a_l, a_r]$ this requires $k = \lceil (a_r - a_l)/(2\sqrt{\mu \max\{\epsilon, b_i\}}) \rceil$ supporting points $p_1, \ldots, p_k$, with $p_j = a_l + (2j - 1)\sqrt{\mu \max\{\epsilon, b_i\}}$.

Empirically we have chosen $\delta := 2$ in $a_l = -\delta b_i$, $a_r = \delta b_i$, and $\mu := 0.001$ in the current implementation of CIDANE. Parameters δ and μ can be easily adjusted to the quality of the data through corresponding command-line arguments.

5 Pricing for general covers

Similar to the graph introduced for the case of single exon spanning reads in Section 4.2 of the main text, we construct a hypergraph $G = (V, E)$. For every segment cover $(\bar{s}_i, \bar{s}'_i, b_i) \in \mathcal{C}$, however, a hyperedge $e_i \in E$ comprises all segments in $\bar{s}_i$ and $\bar{s}'_i$, i.e. $e_i = \{v_j \in V \mid s_j \in \bar{s}_i \vee s_j \in \bar{s}'_i\}$. Generalizing the definition in the main text, $\bar{V}(e_i)$ contains all vertices representing segments located between the last segment s_l of $\bar{s}_i$, and the first segment s_f of $\bar{s}'_i$:

$$\bar{V}(e_i) := \{v_k \in V \mid l < k < f\}.$$

Again, edge weights $w_e : \mathcal{P}(\bar{V}(e)) \mapsto \mathbb{R}$ model the summands on the left-hand side of equation (4) in the main text. Further, we define $\xi(e_i)$ as the set of vertices with associated segments being spanned by either $\bar{s}_i$ or $\bar{s}'_i$:

$$\xi(e_i) := \{v_k \in V \mid f_1 \leq k \leq l_1 \vee f_2 \leq k \leq l_2\},$$

where f_1 (f_2) and l_1 (l_2) are the first and last segments of $\bar{s}_i$ ($\bar{s}'_i$), respectively. Compared to Heaviest Isoform problem defined in the main text, we require an edge to be *strictly induced* by $T \subseteq V$ in order to contribute to the total weight. An edge e is strictly induced by $T \subseteq V$, if the sequence of segments corresponding to vertices in T contains $\bar{s}_i$ and $\bar{s}'_i$ as substrings, i.e.

$$\forall v \in e : v \in T \wedge \forall v \in \xi(e) \setminus e : v \notin T.$$

Using the same set of variables, only minor modifications to the ILP formulation given in the main text are necessary to capture the general Heaviest Isoform problem:

$$\begin{aligned}
\max \quad & \sum_{e \in E} \sum_{\bar{V}_j \subseteq \bar{V}(e)} w_{e,j} y_{e,j} & \\
\text{s.t.} \quad & y_{e,j} \geq \sum_{v_i \in e \cup \bar{V}_j} x_i + \sum_{v_i \in \bar{V}(e) \setminus \bar{V}_j} (1 - x_i) + \sum_{v_i \in \xi(e) \setminus e} (1 - x_i) + & \\
& \quad - |\bar{V}(e)| - |\xi(e)| - 1 & e \in E, \bar{V}_j \subseteq \bar{V}(e) \\
& y_{e,j} \leq x_i & e \in E, \bar{V}_j \subseteq \bar{V}(e), v_i \in e \cup \bar{V}_j \\
& y_{e,j} \leq 1 - x_i & e \in E, \bar{V}_j \subseteq \bar{V}(e), \\
& & v_i \in \{(\xi(e) \setminus e) \cup (\bar{V}(e) \setminus \bar{V}_j)\}
\end{aligned}$$

6 The Heaviest Isoform Problem is NP-complete

Proof. We devise a polynomial-time reduction from the NP-complete INDEPENDENT SET (IS) problem. Given a graph $G = (V, E)$ and an integer K, the IS problem asks whether there exists a subset of at least K vertices none of which are connected by an edge in E.

From G we construct a weighted graph G' by introducing, for every vertex $v \in V$, a duplicate vertex v' and by connecting v and v' by an edge $e = (v, v')$ of weight $w_e = 1$. To every edge in E we assign weight -1. Then there exists an induced subgraph of G' of weight at least K iff there exists an independent set in G of size at least K: Given an independent set I, the subgraph induced by the vertices in I and all their duplicates has weight $|I|$. On the other hand, let a subgraph of G' of weight at least K be induced by a vertex set S. For every induced edge of weight -1 we arbitrarily remove one of its two vertices from S, inducing at most one edge of weight 1 less. The resulting set S' induces a subgraph of weight at least the weight of the subgraph induced by S and does not contain any edges of weight -1. Thus, set $V \cap S'$ is independent and contains at least K vertices. □

7 Space of y-variables

Let f, $\check{\ell}$, $\hat{\ell}$, h, Δ^{in}, and Δ^{out} be defined as in Section 3 and let g be the length of the unsequenced part of a cDNA fragment, i.e. $g := f - 2h$. According to the definition of the adjusted segment cover length (1), we only have to consider isoforms t with

$$\max_{\check{\ell} \le f \le \hat{\ell}} \{\ell_{t,c}(f)\} > 0\,.$$

Therefore, we require

$$\max_{\check{\ell} \le f \le \hat{\ell}} \{\Delta^{out} + h - f + 1\} = \Delta^{out} + h - \check{\ell} + 1 > 0\,,$$

and

$$\max_{\check{\ell} \le f \le \hat{\ell}} \{g + h - \Delta^{in} + 1\} = \hat{\ell} - h - \Delta^{in} + 1 > 0\,.$$

Given isoform t whose sequence of segments contains $\bar{s}' \cdot S_y \cdot \bar{s}''$ as substring, where S_y has transcript sequence length β. Assume $\bar{s}'$ spans positions $[l_1 \mathinner{..} l_2]$ with left- and rightmost read starting positions $(lpos_1, rpos_1)$ and $\bar{s}''$ spans positions $[k_1 \mathinner{..} k_2]$ with left- and rightmost read starting positions $(lpos_2, rpos_2)$. We define the terms $\Delta_1^l, \Delta_2^l, \Delta_1^k$ and Δ_2^k as follows:

$$\Delta_1^l := l_2 - lpos_1, \quad \Delta_2^l := l_2 - rpos_1, \quad \Delta_1^k := lpos_2 - k_1 \quad \text{and} \quad \Delta_2^k := rpos_2 - k_1.$$

Note that these values are the same for all isoforms of the given structure, independent of the segments preceding $\bar{s}'$ and following $\bar{s}''$. Since

$$\Delta^{out} = \Delta_1^l + \beta + \Delta_2^k \quad \text{and} \quad \Delta^{in} = \Delta_2^l + \beta + \Delta_1^k\,,$$

we only consider elements in $\bar{V}(e)$ with

$$\beta > \check{\ell} - h - 1 - \Delta_1^l - \Delta_2^k \quad \text{and} \quad \beta < \hat{\ell} - h - \Delta_2^l - \Delta_1^k + 1\,,$$

which we obtain through an efficient splicing graph based backtracking scheme.

References

[1] Heber, S., Alekseyev, M., Sze, S.H., Tang, H., Pevzner, P.A.: Splicing graphs and EST assembly problem. Bioinformatics (Oxford, England) **18 Suppl 1**, 181–188 (2002)

[2] Li, J.J., Jiang, C.-R., Brown, J.B., Huang, H., Bickel, P.J.: Sparse linear modeling of next-generation mRNA sequencing (RNA-Seq) data for isoform discovery and abundance estimation. Proceedings of the National Academy of Sciences **108**(50), 19867–19872 (2011)
